# Enhanced Performance by Zn-Substitution in Biphasic P2/P3–Na_0.75_Mn_0.68_Ni_0.25_Zn_0.07_O_2_

**DOI:** 10.1021/acs.chemmater.6c01041

**Published:** 2026-06-22

**Authors:** Yingling Liao, Rachel Gordon, Oxana V. Magdysyuk, Aaron B. Naden, Maximillian G. Stanzione, Pontus Törnblom, Moritz Hirsbrunner, Laurent C. Duda, A. Robert Armstrong

**Affiliations:** † EaStCHEM, School of Chemistry, 7486 University of St Andrews, St Andrews, Fife KY16 9ST, United Kingdom; ‡ The Faraday Institution, Quad One, Harwell Science and Innovation Campus, Didcot OX11 0RA, United Kingdom; § Department of Physics and Astronomy, Division of Molecular and Condensed Matter Physics, 8097 Uppsala University, Uppsala S-75120, Sweden

## Abstract

Zn-substituted Na_0.75_Mn_0.68_Ni_0.25_Zn_0.0_7O_2_ has been synthesized in P3, P2, and composite P2/P3 structures and compared with unsubstituted Na_0.7_Mn_0.75_Ni_0.25_O_2_ analogues as positive electrodes for sodium-ion batteries. The synthesis temperature was shown to provide a means of controlling the phase ratio of P2 and P3 phases. Powder diffraction measurements, high-resolution transmission electron microscopy (TEM), and selected area electron diffraction (SAED) revealed that Zn substitution enhanced the ordering of the transition metal (TM) layers. Electrochemical studies combined with XAS measurements showed that after Zn substitution, Ni activity was enhanced, while the irreversible activity of O and Mn was suppressed. Structural transformations were suppressed, and the reversibility of Zn-substituted samples on cycling was improved. Among the Zn-substituted samples, Zn–P2/P3 delivers the best electrochemical performance with an initial capacity of 121 mAh g^–1^ at a rate of 25 mA g^–1^ and 90% capacity retention after 100 cycles in half-cells. This work reveals the intrinsic correlation among cation doping, synthesis conditions, and crystal phase compositions but also provides a reliable strategy for designing high-stability composite layered cathode materials for sodium-ion batteries.

## Graphical abstract

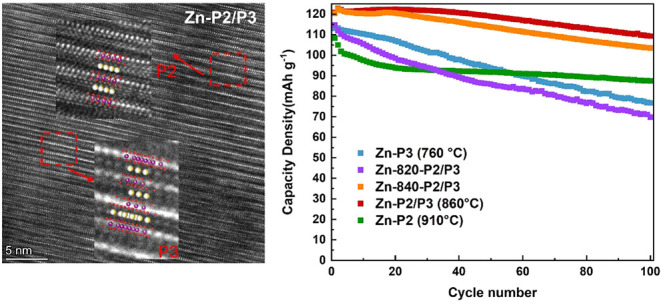

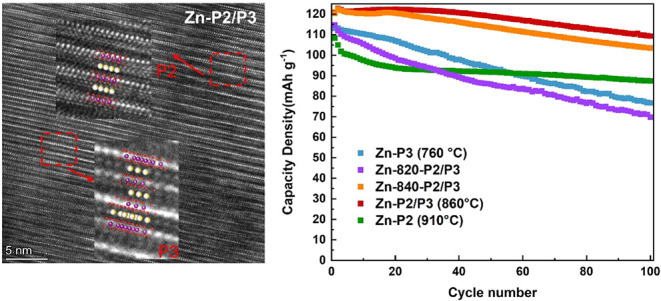

## 1 Introduction

As society develops, there is ever-increasing demand for sustainable energy storage solutions. Lithium-ion batteries (LIBs) are the current market leader, but they are unlikely to meet the growing demand because of the volatile price of lithium and its uneven geographical distribution.^1^ By contrast, sodium is abundant, evenly distributed, and both cheaper and less prone to price variation than lithium.^2^ As such, sodium-ion batteries (NIBs) are considered one of the most promising options for electrical energy storage systems (EES).^3,4^ Among the different classes of positive electrode materials for NIBs, one of the most promising candidates, manganese-based sodium layered oxides Na_*x*_TMO_2_ (where TM = transition metal, 0 < *x* < 1), has attracted great interest because of elemental abundance and high energy densities enabled by the use of both cationic and anionic redox reactions.^5^ These materials can form different polymorphs, which include O3 (space group – *R*3̅*m*), P3 (*R3m*), and P2 (*P6*_3_ */mmc*) type structures, which are classified according to the Na coordination and the number of different oxygen layers in the unit cell.^3,6^ The Na^+^ ions can occupy either prismatic (P) or octahedral (O) sites, and the number (i.e., 2 or 3) represents the number of repeated layers (A, B, or C) within the unit cell, as illustrated in Figure 1.^1^

**Figure 1:**
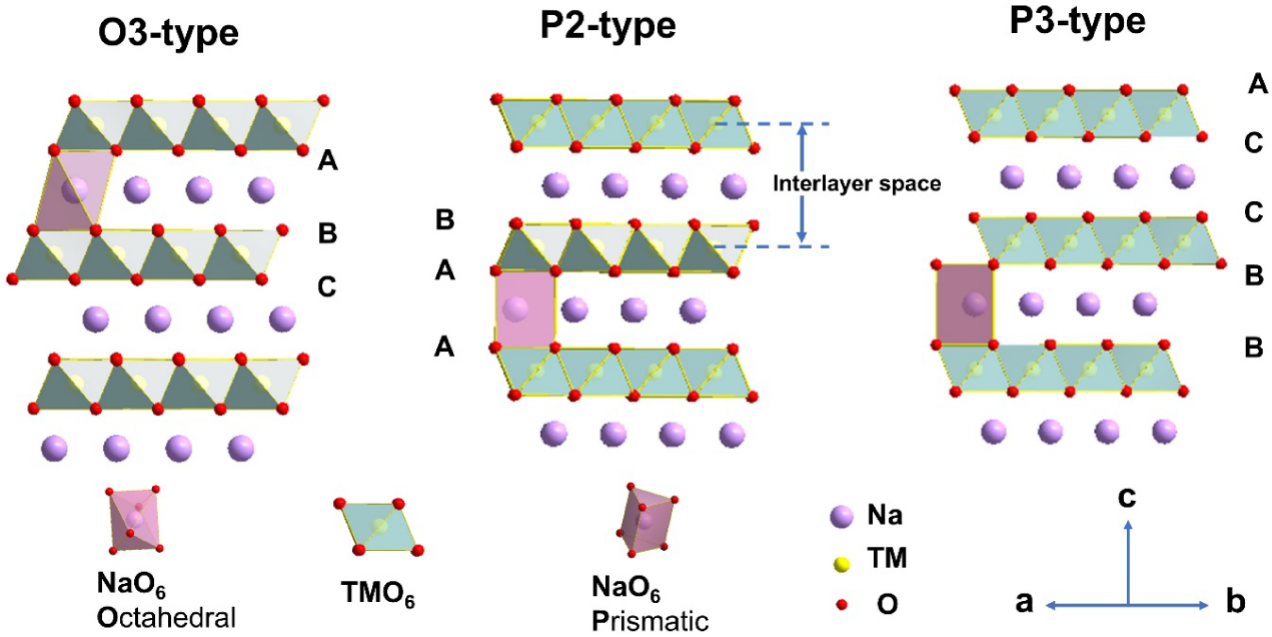
Illustrations of the crystal structures for O3, P2, and P3 type layered oxides. Green polyhedra represent TMO_6_ octahedra, and purple polyhedra represent NaO_6_ polyhedra. Na, TM, and O are shown by purple, yellow, and red spheres, respectively.

Comparatively, O3-type materials typically contain more Na (>0.8) and can therefore deliver higher reversible specific capacities but generally suffer from limited cycling stability owing to complex phase transformations and poor rate capability as a result of bottlenecks for Na-ion diffusion.^7^ On the other hand, P-type materials typically show better electrochemical performance in terms of rate capability than O-type materials because Na^+^ ions can diffuse between prismatic sites without any bottlenecks and possess larger interlayer separation, enabling faster kinetics. There have been extensive studies performed on single P3 and especially P2–Na_*x*_TMO_2_.^3,8–10^ However, relatively few reports have focused on biphasic layered materials. These multiphase materials (such as P3/O3, O3/P2, P2/P3) may show enhanced electrochemical properties and rate capability.^1,11,12^ For example, Huang et al. reported that biphasic P3/P2- and triphasic P3/P2/O3′-Na_0.62_Li_*x*_Mn_0.66_Ni_0.17_Co_0.17_O_2_ (*x* = 0 or 0.18) demonstrated 57.5% and 78% discharge capacity retention after 50 cycles at 0.2 C, respectively. These materials also exhibited better rate capability (around 80 mAh g^–1^ and 110 mAh g^–1^ at 5 C discharge rate, respectively) than single-phase P3–Na_0.62_Mn_0.66_Ni_0.17_Co_0.17_O_2_ (54.1% capacity retention under the same cycling conditions, 73 mAh g^–1^ at 5 C).^13^ The intergrowth of biphasic materials may deliver a positive effect on electrochemical properties, but the mechanism for this improvement is complex and poorly understood.

In addition, cation substitution can introduce positive effects in improving electrochemical performance. Previous research has shown that substitution with high Pauling electronegativity, electrochemically inactive elements like Mg^2+^ (1.31), Ti^4+^ (1.54), and Zn^2+^ (1.65) enables the formation of Mg–O, Ti–O, and Zn–O bonds, thus creating O 2*p* nonbonding orbitals and enhancing anionic redox.^14–18^ For example, Bai et al. and Linnell et al. both found that Zn substitution led to enhanced anionic redox in P2–Na_2/3_Mn_7/9_Zn_2/9_O_2_ and P3–Na_0.67_Mn_0.9_Zn_0.1_O_2_, respectively, which triggered anionic redox without O_2_ release and delivered a reversible capacity of 195 mAh g^–1^ between 1.5 and 4.2 V at a rate of C/10 in P2–Na_2/3_Mn_7/9_Zn_2/9_O_2_. In P3–Na_0.67_Mn_0.9_Zn_0.1_O_2_ 96% of the initial capacity was maintained after 30 cycles between 1.8 and 3.8 V, resulting from high structural stability.^15,19^ Similarly, Konarov et al. demonstrated that Zn-substituted P2–Na_2/3_[Mn_0.7_Zn_0.3_]­O_2_ delivered a high discharge capacity of 190 mAh g^–1^ and 80% capacity retention after 200 cycles at 26 mA g^–1^, between 1.5 and 4.6 V.^20^ Zn substitution has been found to reduce the distortion degree of the Ni–O octahedra and improve the reversibility of the structure in P2–Na_0.66_Ni_0.33_Mn_0.67_O_2_. As a result, in P2–Na_0.66_Ni_0.26_Zn_0.07_Mn_0.67_O_2_, Zn substitution decreased the polarization between 3.8 and 4.1 V and smoothed the voltage plateaus with 93.1% capacity retention after 10 cycles at 12 mA g^–1^ between 2.2 and 4.3 V.^21^ While Zn-substituted P2- and P3-type sodium layered oxides exhibit improved cycling stability and stable structures, the effects of Zn substitution in biphasic P2/P3 materials have not been explored to date.

Consequently, to combine the benefits of both P2 and P3 phases and the effects of Zn substitution, this work studied Zn-substituted P2/P3–Na_0.75_Mn_0.68_Ni_0.25_Zn_0.07_O_2_ (referred to as Zn–P2/P3) materials prepared at a range of temperatures spanning the range 700 °C (P3) to 900 °C (P2). Samples prepared at 820, 840, and 860 °C exhibited a progressive increase in P2-phase content as a function of synthesis temperature. Zn-substituted P2–Na_0.75_Mn_0.68_Ni_0.25_Zn_0.07_O_2_ (Zn–P2) and P3–Na_0.75_Mn_0.68_Ni_0.25_Zn_0.07_O_2_ (Zn–P3) were investigated to help inform understanding of the electrochemically induced structural changes of the biphasic P2/P3 materials. Other substitution levels containing 5% and 10% Zn were also prepared, but since their performance was inferior to that of 7% Zn, they are not considered here. In addition, we prepared the unsubstituted P2, P3, and P2/P3–Na_0.75_Mn_0.75_Ni_0.25_O_2_ (denoted herein as NMN-P2, NMN-P3, and NMN-P2/P3, respectively) to gain an in-depth understanding of the effects of Zn substitution.

Using a combination of techniques, including cyclic voltammetry (CV), transmission electron microscopy (TEM), and powder diffraction, the Zn-substituted materials were found to form superlattices, enhancing Ni redox while suppressing but also stabilizing the anionic redox behavior, thus leading to excellent cycling stability with high-capacity retention. This, therefore, provides valuable insight into the realization of the next generation of high-performance Na-ion batteries.

## 2 Experimental Section

### 2.1 Material Synthesis

Stoichiometric amounts of NaNO_3_ (99.0%, Alfa Aesar), Mn­(NO_3_)_2_•6H_2_O (98+%, Alfa Aesar), Ni­(NO_3_)_2_•6H_2_O (99.0%, Acros Organics), and Zn­(NO_3_)_2_•6H_2_O (≥99.0%, Sigma-Aldrich) were dissolved in 20 mL deionized water to make solution A. Then stoichiometric amounts of C_6_H_8_O_7_•H_2_O (citric acid, chelating agent) were dissolved in 20 mL deionized water to give solution B. Next, solution B was added dropwise to solution A under stirring and left to stir for 2 h at room temperature before the temperature was increased to 80 °C overnight. The resulting powders were first heated at 500 °C for 5 h. Subsequently, powders were heated at different temperatures between 700 and 900 °C for 3 h in order to produce different phases (i.e., P2, P3, or P2/P3 biphasic) and cooled to 50 °C at a rate of 5 °C min^–1^. The as-synthesized samples were stored in an argon-filled glovebox.

### 2.2 Structural Characterization

Laboratory powder X-ray diffraction (PXRD) patterns were collected in Debye–Scherrer geometry on a STOE STADI/P diffractometer with a Mythen (DECTRIS) detector, using Mo Kα_1_ radiation (λ = 0.70930 Å). Data were collected at room temperature in the 2θ range of 4.0–53.0° over 50 min per scan with a step size of 0.015° and a time per step of 0.92 s. Samples were loaded into glass capillaries (0.5 mm diameter) in an argon-filled glovebox. To assemble an *operando* SPXRD cell, a 5 mm diameter hole was drilled in the center of a CR2032 positive coin cell case, CR2032 negative cell case, and the spacer. After assembling the cell, one Kapton foil was used on each side of the cell to seal it with Epoxy H70E (Epoxy Technology). The SPXRD and *operando* SPXRD were performed on the I11 diffractometer (l = 0.824725 Å) at Diamond Light Source, UK. Powder neutron diffraction (PND) was performed on the Polaris diffractometer^22^ at ISIS Neutron and Muon Source, Rutherford Appleton Laboratory, UK. Diffraction data were refined via the Rietveld method using Topas Academic V6.19.^23^ Scanning electron microscopy (SEM) and energy-dispersive X-ray spectroscopy (EDS) analyses were performed on the as-synthesized materials using a JEOL JSM-IT800 equipped with a JEOL DrySD EDX spectrometer. Scanning transmission electron microscopy (STEM) and selected area electron diffraction (SAED) were used to investigate the spatial distribution of the two phases on the nanoscale. Samples for STEM were prepared in the same way as the electrodes prepared for electrochemical measurements, followed by cross-sectioning using a focused ion beam (FIB) on an FEI Scios with bulk milling at an acceleration voltage of 30 kV, followed by final polishing at 5 kV. STEM measurements were performed on an FEI Titan Themis operated at 200 kV, equipped with a CEOS DCOR probe corrector, a SuperX energy-dispersive X-ray spectrometer (EDX), and a 4k × 4k CETA CMOS camera.

### 2.3 Electrochemical Measurements and *Ex-Situ* Characterization

Electrodes were prepared by mixing 80 wt % of the active material with 10 wt % super C65 carbon (Imerys) and 10 wt % Solef 5130 binder (modified polyvinylidene fluoride (PVDF), Solvay). Slurries were stirred in *n*-methyl-2-pyrrolidone (NMP, Alfa Aesar, 99.5%) for 4 h before being cast on aluminum foil (Advent Research) using a doctor blade. After drying at 80 °C for 2 h in a dry room, 13 mm disc electrodes were punched, dried overnight at 80 °C under vacuum, and transferred into an argon-filled glovebox (H_2_O and O_2_ lower than 1 ppm). CR2032 coin cells were assembled, which were made up of a positive disk electrode, sodium metal as a counter/reference electrode, a glass fiber separator (Whatman, GF/F) soaked with electrolyte (1 M NaPF_6_ in ethylene carbonate/diethyl carbonate (1:1 v/v%), Kishida). For the pouch cell measurements, an electrode composition of 92% active material, 5% carbon, and 3% PVDF was used. A frequency range of 0.01 Hz–10 kHz was used to collect EIS spectra, and cells were run at 25 mA g^–1^. EIS fitting was done using RelaxIS 3. Electrochemical measurements were performed in a temperature-controlled chamber at 30 °C using either a BTS-4000 Neware battery tester or a Bio-Logic BCS-805 modular battery testing system.

### 2.4 For *Ex-Situ* Characterization

80 wt % active material was mixed with 20 wt % super C65 carbon in an argon-filled glovebox. The working electrodes were cycled in Swagelok cells. After cycling, Swagelok cells were disassembled in an argon-filled glovebox. The electrode material was then washed 3 times with dry dimethyl carbonate (DMC, Sigma-Aldrich, ≥99%) and dried under vacuum overnight.

### 2.5 Soft X-ray Spectroscopy

For *ex situ* soft X-ray absorption spectroscopy (SXAS) and resonant inelastic X-ray scattering (RIXS), coin cells were assembled as described above except that Solupor membranes replaced the glass fiber separator. Manganese L-edge and oxygen K-edge SXAS and RIXS measurements were collected at the C-branch of beamline BL27SU at Spring 8 in Japan.^24^ Bulk-sensitive SXAS spectra were obtained in partial fluorescence yield (PFY) mode and inverse partial fluorescence yield (IPFY) mode, the latter is derived by inverting the O K-edge PFY. All SXAS spectra were normalized by the incident intensity, measured by recording the sample drain current and focusing the mirror current by using picoammeters. Baselines were fitted to the mostly flat regions below any absorption peaks and subsequently subtracted from each spectrum. Further normalization was carried out by setting the low-energy region before any absorption peaks to zero and the postedge region after any absorption features to unity.

## 3 Results and Discussion

### 3.1 Structural Characterization

#### 3.1.1 Powder X-ray Diffraction

Powder X-ray diffraction (PXRD) patterns were fitted to ideal P2 and P3 structural models, and fitted profiles of the as-synthesized Zn-substituted materials are shown in Figure 2, while those of the unsubstituted materials are shown in Figure S1. Figure 2a shows the refined PXRD pattern of Zn-substituted Zn–P3 showing the major diffraction peaks corresponding to an ideal P3-type structure (space group *R3m*) with characteristic peaks (107) and (108). As the synthesis temperature was raised, the ratio of P2:P3 components progressively increased. In the case of Zn–P2/P3, as the temperature was increased from 820 to 840 and 860 °C, the quantity of the P2 phase increased from 41.7% to 56.5%, reaching 83.8% at 860 °C. Figure 2b–2d show the patterns from Zn–P2/P3 at 820 °C, 840 °C, and 860 °C, respectively. Figure 2e shows the fitted PXRD pattern for Zn–P2 (space group *P6*_3_ */mmc*) with the unique (106) diffraction peak indicated. In general, from Rietveld refinement (details displayed in Tables S1–S8), NMN-P2/P3 and Zn–P2/P3 synthesized at 860 °C contain similar percentages of P2 and P3 components (88% P2 and 12% P3 in NMN-P2/P3, 84% P2 and 16% P3 in Zn–P2/P3). The *a* lattice parameter and cell volume are enlarged in Zn-substituted phases due to the larger ionic size of Zn^2+^, as detailed in Table S10. By contrast, the *c* lattice parameter decreased for Zn-substituted samples, which may be attributed to the larger Pauling electronegativity of Zn^2+^. The P2-containing Zn-substituted materials show additional reflections not observed for the unsubstituted materials, corresponding to superlattice reflections arising from the long zigzag model. In addition, ordering of the TM layer is to be expected in this case since the ratio of smaller Mn^4+^ to larger M^2+^ (M = Ni, Zn) is close to 2:1, which favors a honeycomb arrangement. This driving force is absent in the unsubstituted material. Na^+^/vacancy ordering (so-called long zigzag ordering) has been widely reported in P2 layered oxides, but this is absent in Na_*x*_Mn_0.75_Ni_0.25_O_2_ as reported by Pfeiffer et al. and Gutierrez et al. since the Mn^3+^ introduces defects into the Ni–Mn interplane charge order that in turn disrupts the ordering within the Na-layers.^25,26^

**Figure 2:**
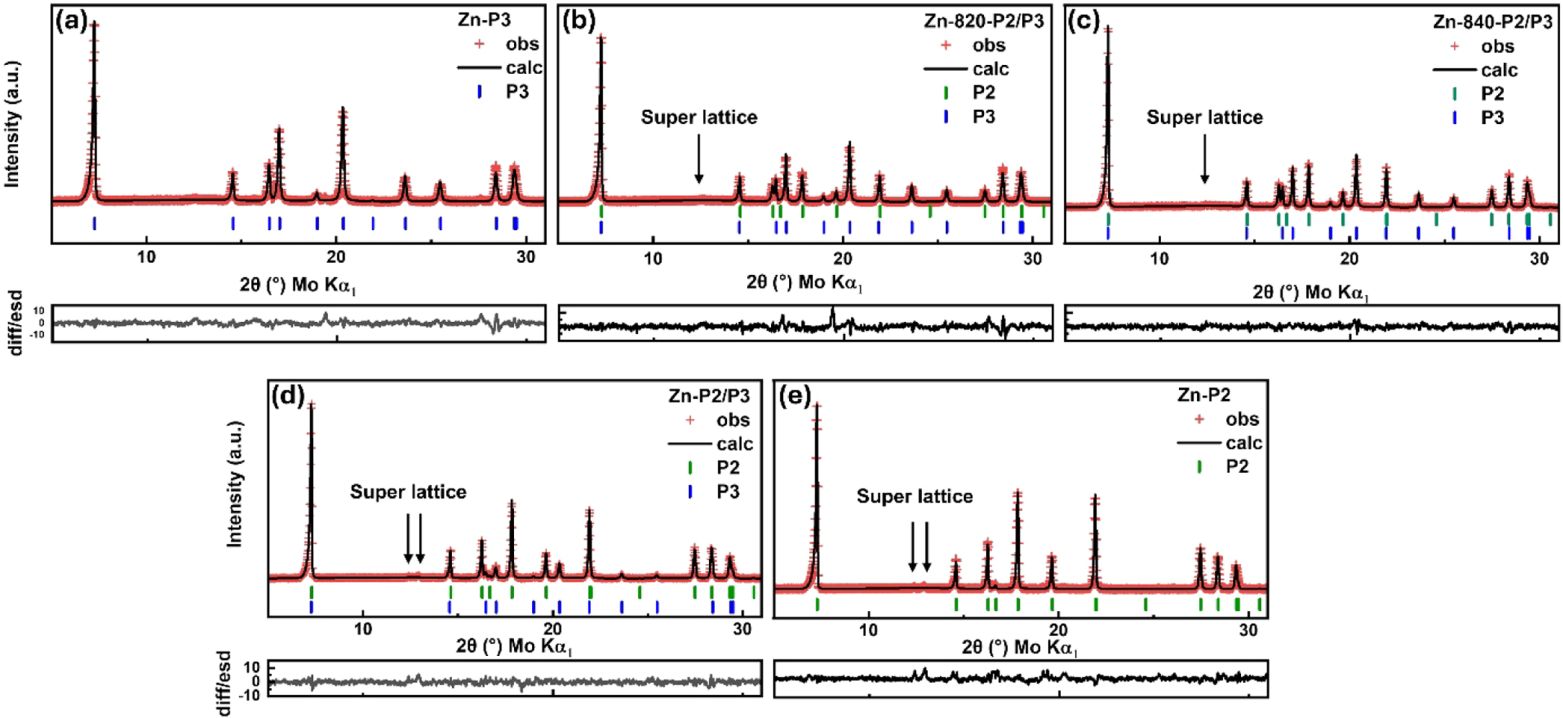
Laboratory X-ray profile fits of as-synthesized Zn-substituted materials: (a) Zn–P3, (b) Zn-820-P2/P3 (calcined at 820 °C), (c) Zn-840-P2/P3 (calcined at 840 °C), (d) Zn–P2/P3 (calcined at 860 °C), and (e) Zn–P2. Observed data points are shown in red, with the fitted profile in black. Green and blue tick marks indicate allowed reflections for the P2 and P3 phases, respectively.

#### 3.1.2 Superlattice Identification

In order to characterize the ordering behavior observed above, samples were further studied using synchrotron X-ray powder diffraction (SPXRD) and powder neutron diffraction.

Initial refinement of the powder neutron diffraction data for Zn–P2/P3 used primitive unit cells for both P2 (*P6*_3_ */mmc*) and P3 (*R3m*) phases. A satisfactory fit (R_wp_ = 8.88%) was obtained, but additional reflections were readily apparent, derived from superlattice formation. These superlattice reflections derived from both sodium-vacancy ordering and especially TM ordering are pronounced as displayed in Figures 3 and S2 and Table S9. In addition, many of the more intense reflections from the majority P2 phase are poorly fitted, suggesting a distortion from the ideal hexagonal structure. This was confirmed by synchrotron powder diffraction measurements, where the higher instrumental resolution shows clearly resolved peak splitting (as shown in Figure S2). As a result, a distorted unit cell (space group *C222*_1_), which has previously been reported in related materials, was adopted.^25^ The quantity of the P3 phase in the sample prepared for neutron diffraction was insufficient (8%) to obtain accurate refined structural parameters and to confirm the formation of a superlattice. As such, Table S9 only details the lattice parameters for this phase. Refined sodium occupancies show good agreement with expected values with no evidence for Zn occupancy in the sodium layer.

**Figure 3:**
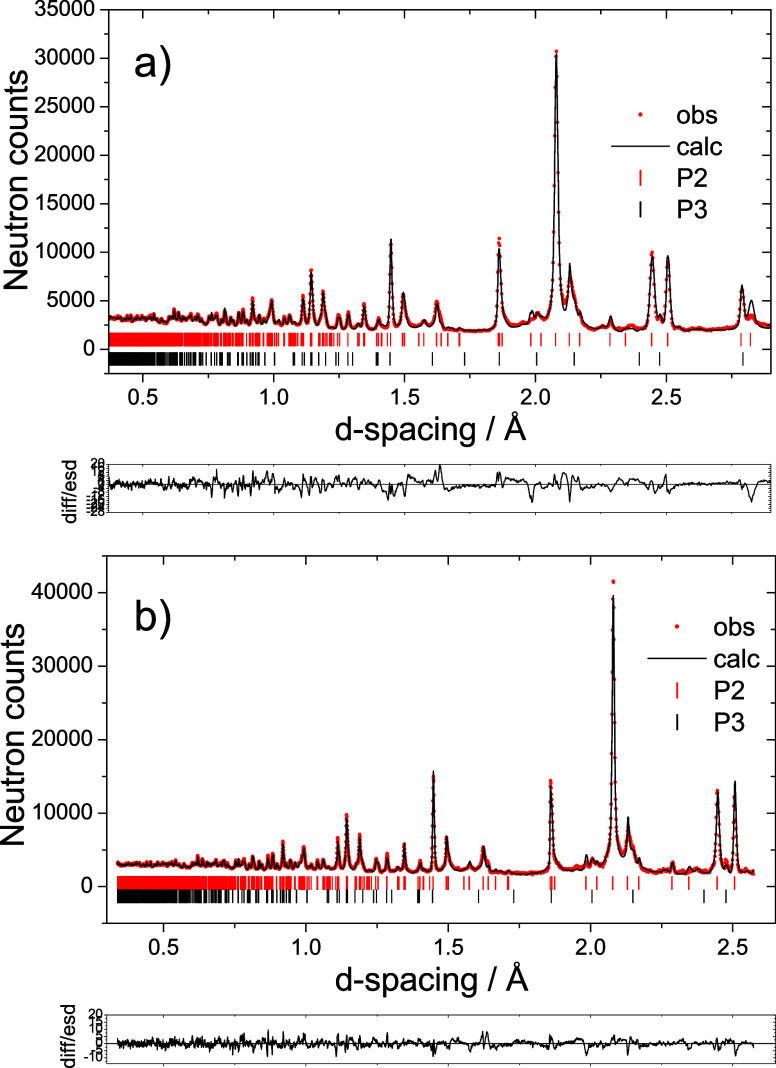
Profile fits for Rietveld refinements of Zn–P2/P3 for (a) Bank 4 (90°) and (b) Bank 5 (backscattering) powder neutron diffraction data collected on the Polaris diffractometer, adopting space group *C222*_1_ for the P2 phase and space group *R3m* for the P3 phase, respectively. Observed data points are shown in red, with the fitted profile in black. Tick marks indicate allowed reflections.

### 3.2 Electron Microscopy Analysis

Scanning electron microscopy (SEM) combined with energy-dispersive spectroscopy (EDS) mapping was carried out to explore the morphology and elemental distribution of Zn-substituted and unsubstituted materials, as shown in Figure 4. Comparatively, Zn substitution does not appear to alter the morphology of the particles, and the temperature is the main factor that causes a change in the morphology of the materials, as the higher synthesis temperature produces larger particle sizes. The EDS elemental maps reveal a homogeneous distribution of the elements, with no clusters of Zn, Na, or O observed, which indicates that Zn has been successfully incorporated into the material structure, consistent with the powder diffraction analysis (Figure 2).

**Figure 4:**
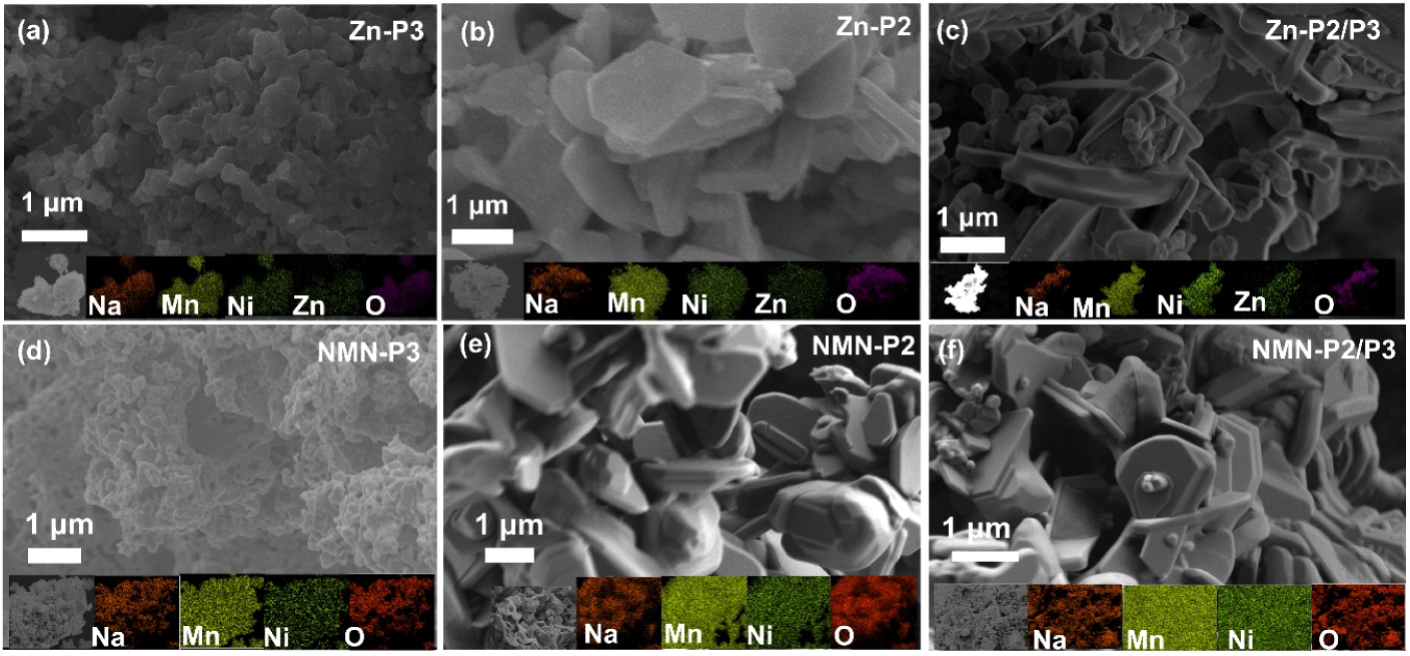
(a–c) SEM images and EDS maps showing Na, Mn, Ni, Zn, and O for Zn-substituted Zn–P3, Zn–P2, and Zn–P2/P3 (prepared at 860 °C). (d–f) SEM images and EDS maps showing Na, Mn, Ni, and O for unsubstituted NMN-P3, NMN-P2, and P2/NMN-P3.

The microstructures of the materials were studied by scanning transmission electron microscopy (STEM) combined with corresponding selected area electron diffraction (SAED) patterns, which are displayed in Figure 5. From the STEM images, it is clear that all materials have complex atomic arrangements with nanoscale intergrowth observed in P2/P3-type materials. The SAED patterns can be indexed into the layered structure along different zone axes, as depicted in Figure 5.

**Figure 5:**
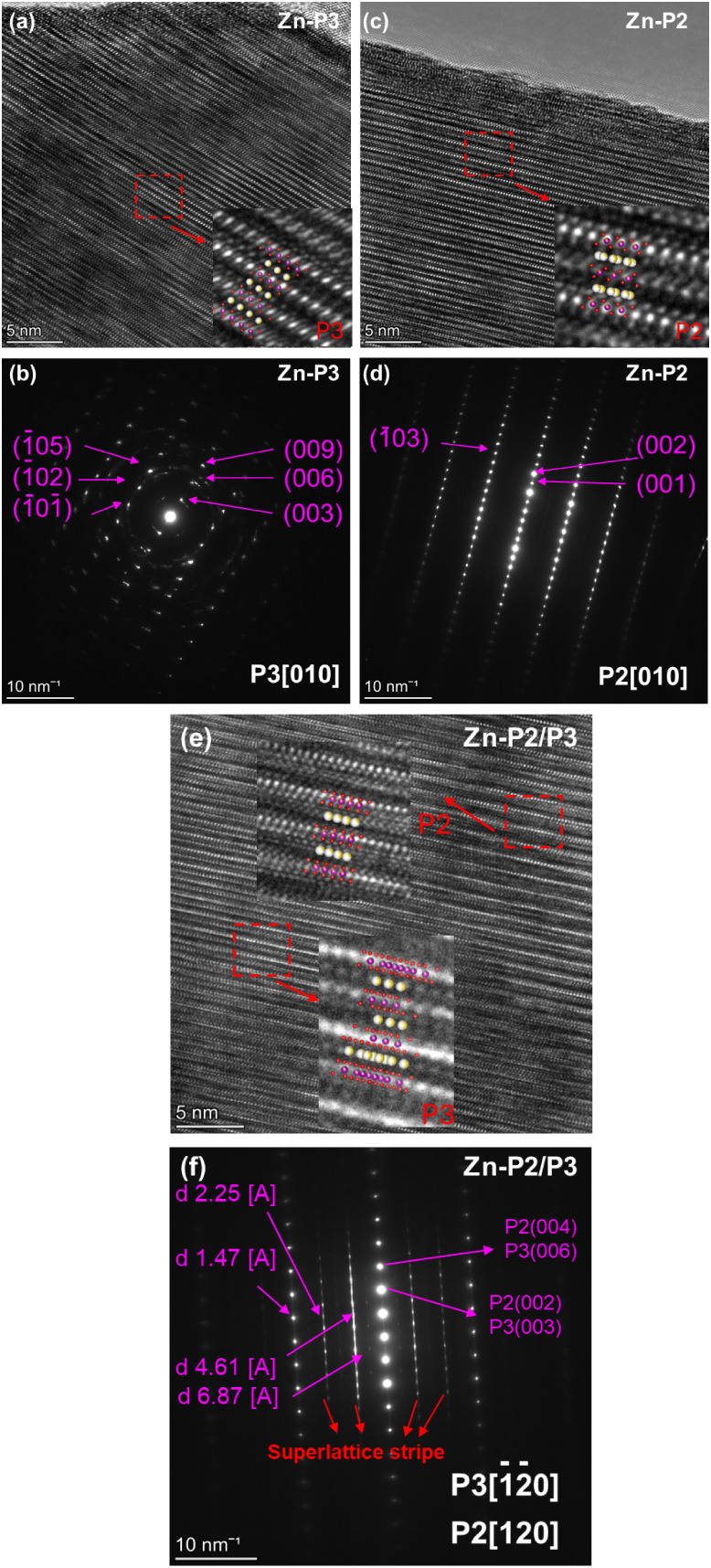
STEM images with magnified phase regions and corresponding selected area diffraction (SAED) patterns of (a–b) Zn–P3, (c–d) Zn–P2, and (e–f) Zn–P2/P3. The zone axis of each phase and some diffraction patterns are labeled in SAED images. The d-space of the main diffraction pattern and the streaks on the same horizontal line are labeled to indicate the superlattice positions in PXRD patterns.

As shown in Figure 5f and Figure S3f, the diffraction patterns of P2 and P3 phases in NMN-P2/P3 and Zn–P2/P3 overlap, caused by the similar unit cell *a* parameters of P2 and P3 phases (in Zn–P2/P3, *a* = 2.8937(3) Å in the P2 phase, and *a* = 2.8893(2) Å in the P3 phase), and also because of the similarity of the interlayer space of P2 and P3 phases (for example, in Zn–P2/P3, the slab thickness is 5.57 Å for the P2 phase and 5.59 Å for the P3 phase), as shown in Table S8.

In the Zn-substituted materials, the structure appears broadly similar to unsubstituted materials but with additional in-plane superlattice reflections in the P2 phase, indicating additional longer-range ordering. This superstructure ordering can be seen as very weak reflections in the laboratory PXRD patterns (Figure 2) but is much more prominent in neutron powder diffraction and synchrotron PXRD as shown in Figure S2 and Figure 3.

Similar reflections can be seen in the corresponding Zn–P2 material, though these are less well-defined than in the Zn–P2/P3 composite (Figures S3 and S4), instead appearing as streaks, pointing to a lower degree of ordering, which is in keeping with the PXRD pattern of Zn–P2 (Figure 2e). No significant streaked reflections were found in Zn–P3; this is probably attributable to the grains of Zn–P3 being too small (Figure 4a) to produce strong enough diffraction and is consistent with the lack of superstructure peaks observed for this phase.

### 3.3 Electrochemical Performance

The electrochemical performance of the Zn-substituted materials is shown in Figure 6, while those of unsubstituted materials are presented in Figure S5. The initial galvanostatic charge–discharge profiles of the undoped and Zn-substituted samples were measured at a current density of 25 mA g^–1^ within the voltage window of 2.2–4.3 V and are presented in Figure S5a–b.

**Figure 6:**
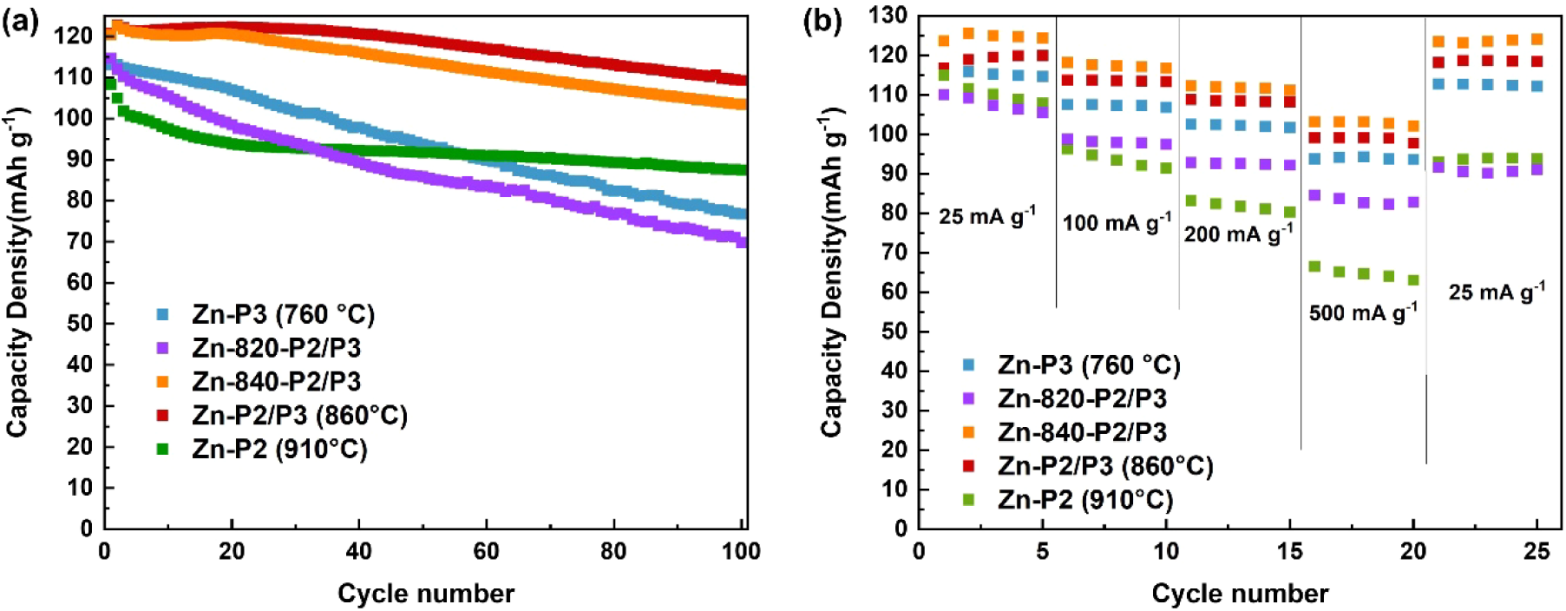
(a) Galvanostatic cycling performance at a rate of 25 mA g^–1^, between 2.2 and 4.3 V for 100 cycles. (b) Rate performance at 25, 100, 200, 500, and 25 mA g^–1^ of Zn–P3, Zn-820-P2/P3, Zn-840-P2/P3, Zn–P2/P3, Zn–P2.

For the undoped NMN series (Figure S5a), NMN-P3, NMN-P2, and NMN-P2/P3 all exhibit high initial discharge capacities, ranging from 127 to 131 mAh g^–1^, with distinct voltage plateaus. Notably, the NMN-P2/P3 biphasic sample shows the smoothest, least polarized profile, indicating improved reaction kinetics compared to the single-phase analogues.

Upon Zn substitution (Figure S5b), the initial discharge capacities are slightly reduced (108–121 mAh g^–1^), yet the voltage profiles become significantly smoother with reduced hysteresis. In particular, the high-voltage sloping region above 3.8 V is less pronounced in the Zn-substituted samples, reflecting the stabilizing effect of Zn on the lattice oxygen redox behavior. Among the unsubstituted materials, NMN-P2/P3 shows the highest capacity retention of 64% after 100 cycles, while NMN-P3 and NMN-P2 show 38%, and 60% capacity retention, respectively. After Zn substitution, the initial discharge capacity decreased slightly for all materials from 131 to 113 mAh g^–1^ for Zn–P3, 127 to 121 mAh g^–1^ for Zn–P2/P3 (with values of 115 mAh g^–1^ Zn-820-P2/P3, and 120 mAh g^–1^ for Zn-840-P2/P3), and 121 to 108 mAh g^–1^ for Zn–P2, perhaps caused by the suppression of anion redox as discussed later. While the Zn-substituted materials deliver a lower initial capacity compared to the parent materials, the capacity retention increased significantly. Zn–P2/P3 annealed at 860 °C showed the highest capacity retention of 90%, with a specific discharge capacity of 109 mAh g^–1^ remaining after 100 cycles, higher than single-phase Zn–P2 (81%) and Zn–P3 (68%). It is reasonable to propose that the excellent cycling stability of Zn–P2/P3 can be attributed to the stable structure, which combines the benefits of single P2- and P3-phases, which are further stabilized by Zn substitution; this will be discussed further. Rate capability was also enhanced in the Zn-substituted P2/P3 composites compared with single phases (Figures 6b and S6), with the best performance from the composite prepared at 840 °C. Moreover, as shown in Figures S5 and S6, Zn-substitution both minimized cell polarization and enhanced energy density. The superior cycling stability and acceptable rate performance of the material prepared at 860 °C meant that this particular material was selected for detailed characterization.

The improved rate performance was further demonstrated qualitatively by comparing the diffusion coefficients derived from the Randles-Ševčík equation as presented in Figure S7.^27^

Figure S7 shows the cyclic voltammetry plots of Zn-substituted materials at different scan rates of 0.025, 0.1, 0.2, 0.3, 0.4, and 0.5 mV s^–1^. The height and area of the redox peaks increased with increasing scan rate, while the anodic peaks shifted to higher voltages and the cathodic peaks shifted to lower voltages, suggesting that Na^+^ ions cannot be completely extracted/reinserted into the structure as a result of the faster scan speed.^28^ The oxidation peaks at around 3.7 V and the reduction peaks at around 3.5 V were selected for quantitative analysis. From the results shown in Figure S7g–h, Zn–P2/P3 exhibits the highest Na^+^ ion diffusion coefficients during both oxidation and reduction processes with the highest slope, which is in good agreement with the rate performance reported in Figure 6b.^29^ The improved sodium transport in Zn–P2/P3 was further demonstrated by electrochemical impedance spectroscopy data (Figure S8). The EIS data of the pristine electrodes in sodium half cells were fitted using an equivalent circuit model. The calculated charge transfer resistance was lowest for the Zn–P2/P3 sample at 425 Ω. The Zn–P3 and Zn–P2 samples had charge transfer resistance values of 514 Ω and 542 Ω, respectively. On charge to 3.5 V, Zn–P2/P3 clearly showed lower impedance, while at the end of charge, impedances for Zn–P3 and Zn–P2/P3 are similar, with Zn–P2/P3 again showing the lowest impedance at the end of discharge.

Preliminary full-cell data were obtained for 3-electrode pouch cells fabricated against commercial hard carbon cycled at C/5 in the voltage window of 1–4.3 V, showing excellent cycling stability (Figure S9).

Cyclic voltammograms of the various materials at a scan rate of 0.025 mV s^–1^ were measured for six cycles to investigate the reversibility of the electrochemical processes during Na^+^ ion extraction/insertion. Plots, normalized to active mass, are displayed in Figure 7. Peaks below 3.0 V primarily arise from the redox processes associated with Mn^3+^/Mn^4+^ (region I), while peaks between 3.0 and 3.8 V correspond to nickel (Ni^2+^/Ni^3+^/Ni^4+^) redox activity (region II), and peaks beyond 3.8 V are typically associated with further nickel redox activity, anion redox, and phase transformation (region III), which were further characterized by XAS, as discussed in Section 2.3 below.^30,31^ From Figure 7, after substituting with Zn, Mn redox is diminished (region I is smaller), which facilitates structural stabilization derived from Mn^4+^ (since Zn^2+^ substitution generates more Mn^4+^ instead of Mn^3+^ for charge balance). As such, lower specific capacity is derived from Mn^4+^ reduction compared with unsubstituted NMN materials. In addition, the redox activity of nickel at lower potential is enhanced, which may also explain the high discharge capacity observed in Zn-substituted materials. In the high-voltage region after Zn substitution, less capacity was obtained on the first cycle, but the high-voltage performance was stabilized by Zn substitution on subsequent cycles, which is in good agreement with the higher cycling stability of all the Zn-substituted materials, Zn–P3, Zn–P2/P3, and Zn–P2 (see Figure 6).

**Figure 7:**
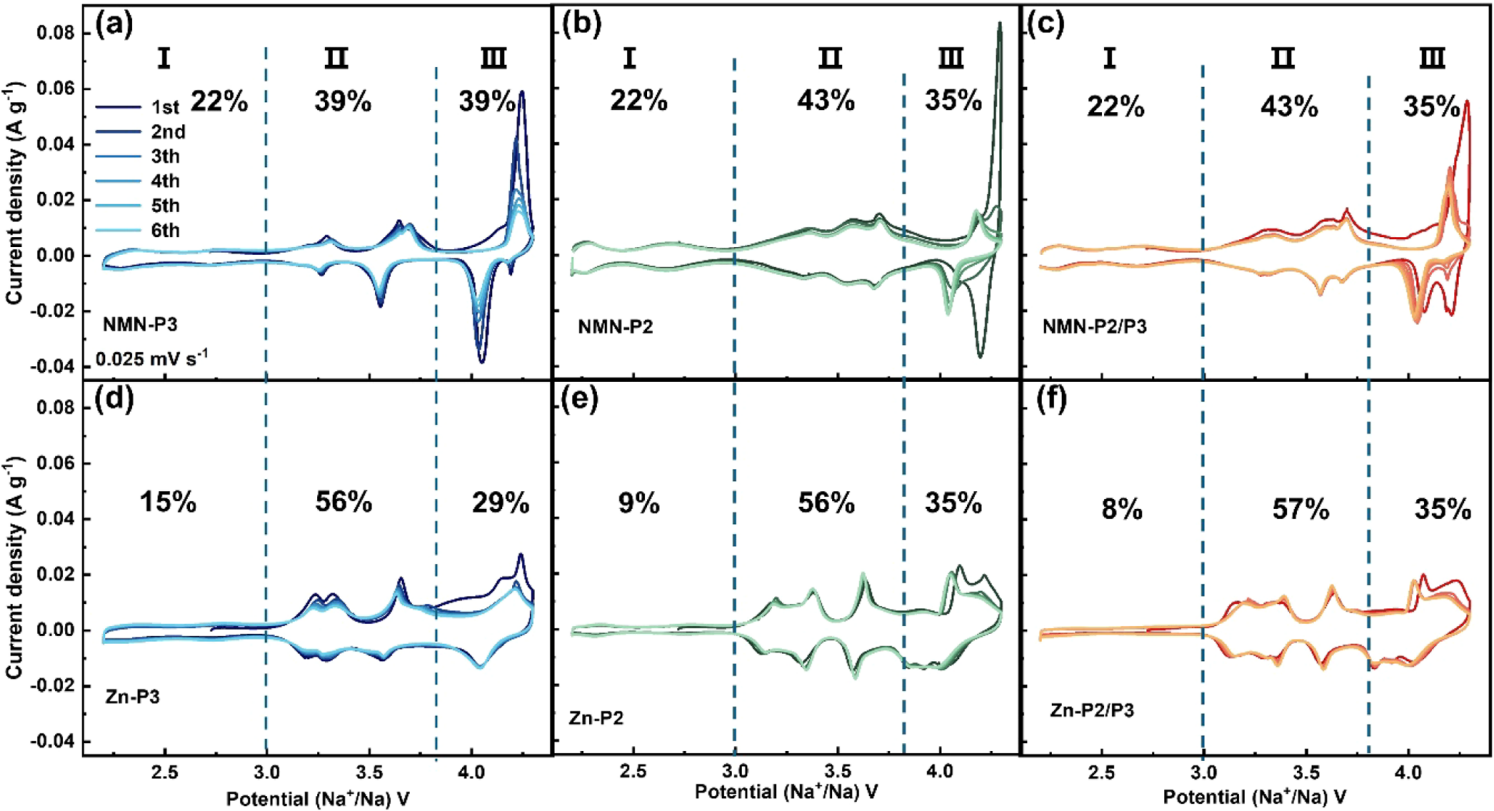
Cyclic voltammograms of (a–f) NMN-P3, NMN-P2, NMN-P2/P3, Zn–P3, Zn–P2, and Zn–P2/P3 with the same scan rates of 0.025 mV s^–1^ for six cycles, between 2.2 and 4.3 V. In the first charge/discharge process, the capacity contribution (discharge capacity divided by the region’s discharge capacity) across regions I, II, and III are labeled.

*Ex situ* Raman spectroscopy provides a useful probe of the TM–O bond changes upon sodiation/desodiation. As demonstrated in Figure S10, NMN-P2/P3 shows complex Na/Mn/Ni–O bonding upon Na^+^ ion insertion/extraction by the change in peak intensities and generation of new peaks. From Figure S10c, though the *ex situ* Raman spectra of Zn–P2/P3 are also complex, the main features of *E*_*g*_ and *A*_1*g*_ peaks are maintained, which highlights the increased structural stability of Zn–P2/P3 compared to NMN-P2/P3. This result is in accordance with the cyclic voltammograms (Figure 7) and the superior electrochemical performance of Zn–P2/P3 (Figures 6 and S6).

In order to understand the structural evolution upon desodiation/sodiation, samples for *ex-situ* laboratory PXRD were prepared by assembling Swagelok cells. As shown in Figure 8 and the *ex-situ* PXRD refinement results displayed in Tables S11–S34, Figures S11–S16, in the unsubstituted P3 phase NMN-P3, a transition to an O′3 phase was observed at the end of charge, corresponding to layer gliding on Na^+^ ion extraction. In the Zn-substituted material Zn–P3, the situation is more complex, with peaks shifting toward an O′3 pattern but retaining some characteristics of P3. This, combined with significant *hkl*-dependent line broadening, indicates the formation of a heavily faulted structure containing both O and P environments. Zn substitution clearly acts to suppress layer gliding, though this may in part be due to reduced sodium extraction on charge. In both cases the host structure was restored at the end of discharge. In the case of single P2 phases, the parent structure was maintained at all states of charge upon Na^+^ ion removal and reinsertion. On sodium extraction, the *a* unit cell parameters (influenced by TM–O bonds) contracted, while the *c* unit cell parameter expanded as a result of the increased Coulombic repulsion between adjacent slabs. In the case of the P2/P3 composite materials, for unsubstituted NMN-P2/P3, a combination of the behavior of NMN-P3 and NMN-P2 was observed on the first cycle. In Zn-substituted materials, similar behavior was noted.

**Figure 8:**
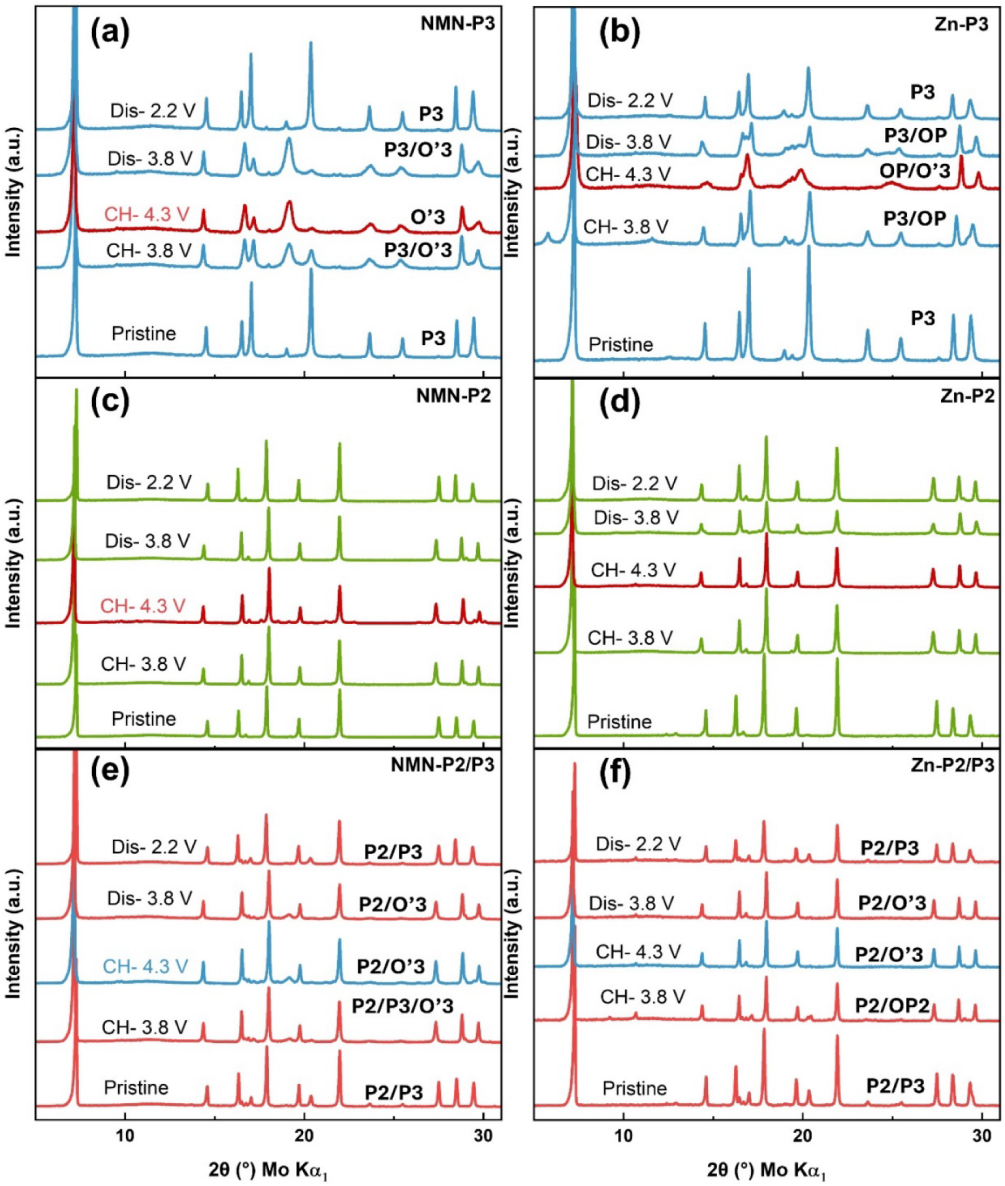
*Ex-situ* PXRD plots of (a) NMN-P3, (b) NMN-P2, (c) NMN-P2/P3, (d) Zn–P3, (e) Zn–P2, and (f) Zn–P2/P3 at pristine, charged to 3.8 V, charged to 4.3 V, discharged to 3.8 V, and discharged to 2.2 V states.

*Ex-situ* PXRD data may not be representative of the true structural evolution since the structures tend to relax over time, which can affect the observed patterns. As such, as a complementary method, *operando* SPXRD was conducted to obtain real-time information on Zn-substituted samples. The first intense peak ((003) in Zn–P3, (002) in Zn–P2, and (002)/(003) in Zn–P2/P3) and related refined results are presented in Figure 9. During the first charge/discharge process, *operando* SPXRD was measured in potentiostatic mode from open circuit voltage to 4.4 V and then back to 2.2 V at a scan rate of 0.05 mV s^–1^. This expanded voltage window was selected to take account of inferior cycling performance in the *operando* setup and to investigate behavior at a high state of charge. As shown in Figure 9a, when first charged to around 4.0 V, the first reflection shifted to a lower angle with slightly increased intensity in all Zn-substituted samples, which results from the expansion in the *c* axis on desodiation. Between 4.0 and 4.4 V, this peak shifted to a lower angle with a newly generated OP peak in Zn–P3 caused by the shorter interlayer space in O-type structures compared to P-type structures. However, the first peak position was maintained after charging to 4.0 V, along with the newly generated OP phase(s) in Zn–P2 and Zn–P2/P3. This result aligns with *ex-situ* PXRD refinement results, which showed a constant *c* cell lattice parameter between 3.8 and 4.3 V. All Zn-substituted samples showed a reversible peak shift, which also is in line with the *ex-situ* laboratory PXRD and *ex-situ* Raman spectroscopy, and further demonstrated the reversible structural evolution.

**Figure 9:**
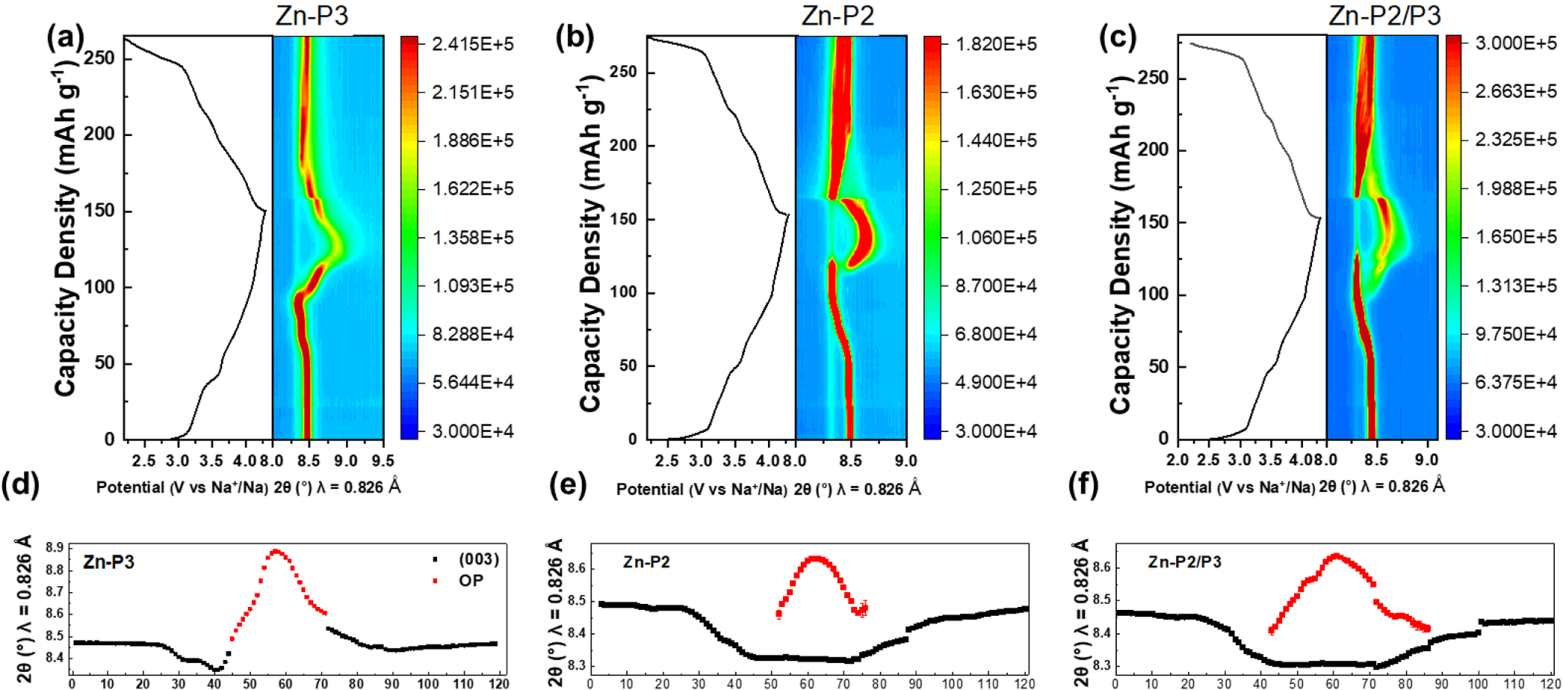
*Operando* SPXRD patterns collected during cyclic voltammogram scans (open voltage: 4.4 V–2.2 V), at a scan speed of 0.048 mV s^–1^ for (a) Zn–P3, (b) Zn–P2, (c) Zn–P2/P3, and (d–f) the shift of the first peak (marked in black) and OP peak (marked in red) in each material.

While PXRD provided information in terms of the long-range average structure, more detailed local structural information (e.g., bond lengths) may be obtained by synchrotron X-ray pair distribution function PDF (xPDF) analysis. As illustrated in Figure 10, the first intense peak corresponds to the first neighbor M–O pairs, which showed a contraction from 1.92 to 1.9 Å in Zn–P3 and Zn–P2/P3 as the average oxidation state of the transition metal increases upon charging, as shown in Table S35. The intensity of the second peak is largely dominated by the first neighbor M–M correlations. This distance also decreased slightly during the charging process. Under the effect of in-plane distortion, the atomic displacement of transition metal atoms is perpendicular to the close-packed hexagonal lattice along the *c* axis, resulting in changes to the M–M distance and interaction with the adjacent layers. This explained the slight contraction upon high-voltage desodiation in *ex-situ* PXRD.^31^ The next group of peaks between 4.3 and 5.1 Å is related to in-plane M–O and M–M interactions.^32^ Zn–P3 presented a smaller contraction in d_3_ (0.08 Å) and d_4_ (0.04 Å) compared to Zn–P2 (0.1 Å, 0.04 Å), revealing a more rigid MO_2_ layer in Zn–P3.^33^ The disappearance of the interlayer M–M distances and low crystallinity after 6.5 Å of fully charged samples revealed the disruption to long-range ordering between the layers.

**Figure 10:**
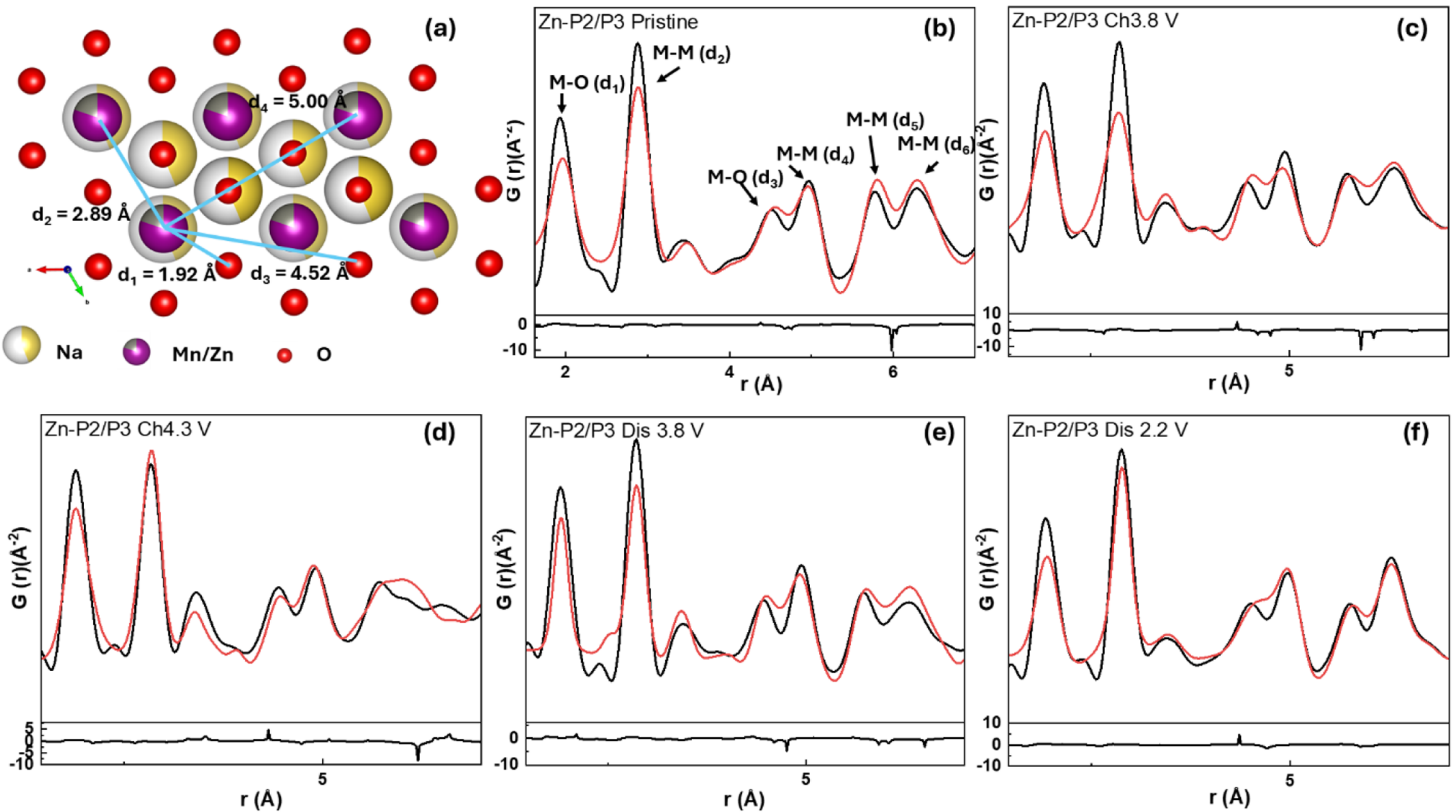
(a) Representation of the labeled atomic radius from different directions. A simplified P3–Na*_x_* TMO_2_ (*x* < 1) model was used. (b–f) *Ex-situ* xPDF with the labeled coordinated bonds of Zn–P2/P3; black line—experimental data, red line—refinement.

### 3.4 Charge Compensation Mechanism

X-ray absorption spectroscopy (XAS) and resonant inelastic X-ray spectroscopy (RIXS) facilitate detailed insight into the electronic structure of the electrode materials. This can reveal changes in the oxidation state of the constituent elements and further pinpoint the participation of oxygen in redox reactions. In Figure 11, XAS spectra are shown for *ex situ* Zn–P2/P3 materials at five different points along the first electrochemical cycle: pristine, charged to 3.8 V, charged to 4.3 V, discharged to 3.8 V, and discharged to 2.2 V (Figure 11). The relevant absorption edges in the soft X-ray regime were investigated, namely, the L-edges of Ni (Figure 11a), Mn (Figure 11b), as well as the O K-edge (Figure 11c), while Zn is known to be electrochemically inactive.^7^ The XAS measurements at the Ni, Mn, and O K-edges for the NMN-P2/P3 sample have been widely reported in the literature for Mn/Ni systems, including our previous work.^34^

**Figure 11:**
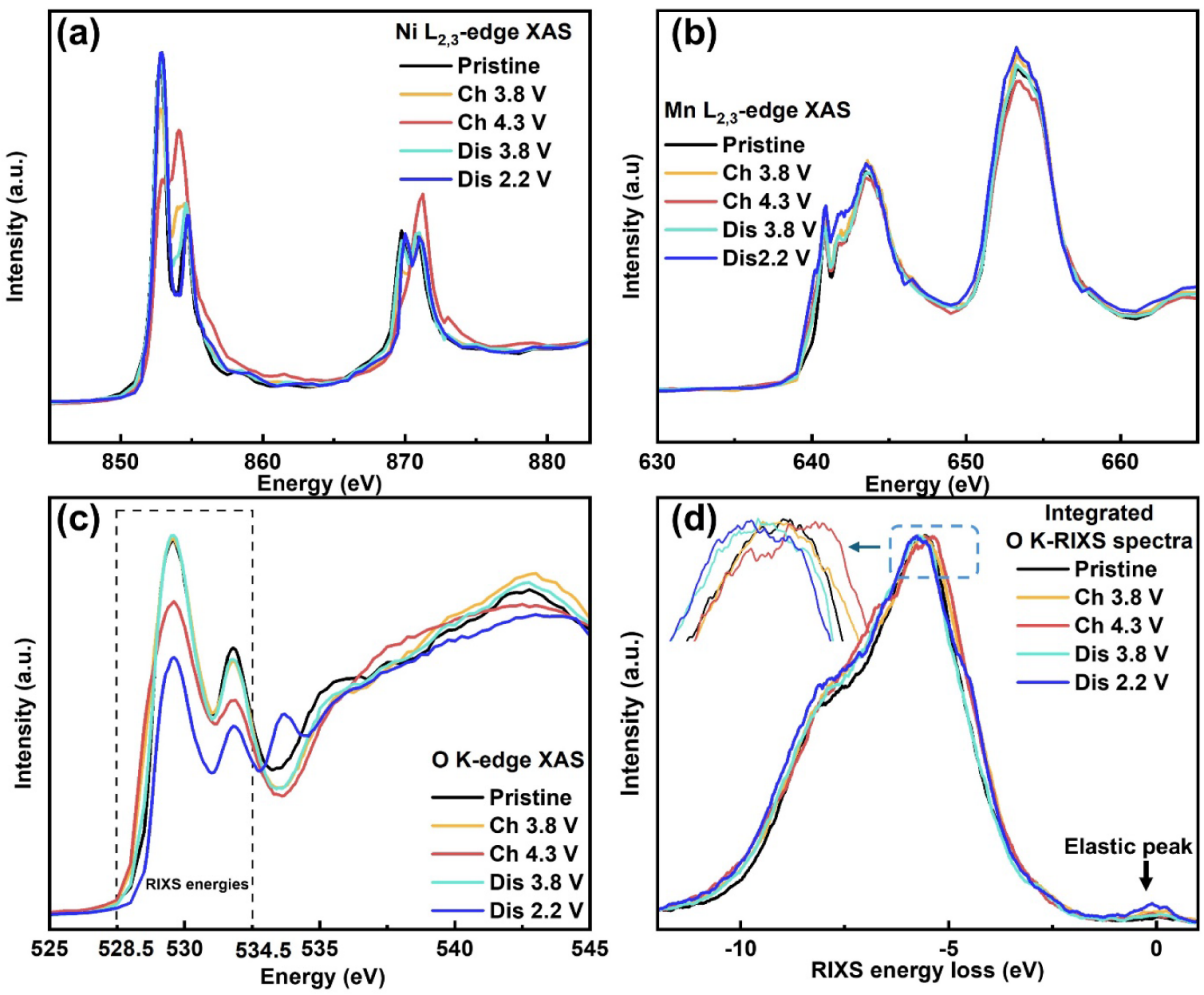
Soft X-ray absorption spectra on the first cycle of Zn–P2/P3 at various absorption edges: (a) Ni L_2,3_-edge, (b) Mn L_2,3_-edge, (c) O K-edge. The spectra were obtained in partial fluorescence yield (PFY) mode and are normalized to after the absorption edge. (d) Integrated O K-edge RIXS spectra over a range of excitation energies, shown for all Zn–P2/P3 materials (530.9 eV–531.5 eV). The spectra have been scaled to the highest intensity to facilitate spectral shape comparisons and are offset for clarity.

Upon charging, the apparent double-peak structure of both the L3 and L2 regions of the Ni XAS spectra shows a reversible shift in spectral weight to higher energies. This signifies a shift of oxidation state of the Ni ions from predominantly Ni^2+^ to more of a Ni^4+^ character.^35^ On the other hand, the Mn L2,3-edge XAS shows that Mn ions are barely affected by electrochemical cycling, as they display only minor spectral changes from a Mn^4+^-like XAS spectrum. However, the Mn L2,3-edge XAS of the Dis 2.2 V sample shows small Mn^3+^ contributions.^35^ This is in line with the expected largely inert behavior of Mn in this material. Turning to the O K-edge in Figure 11c, we focus on the prepeak region (526–532.8 eV), the intensity of which is correlated to the degree of hybridization between the oxygen and neighboring transition metal atoms.^35^ When the neighboring transition metal increases its oxidation number, the hybridization is expected to be stronger as the oxygen compensates for some of the charge.^36^ In the figure, this is seen as a reversible shift in the absorption edge onset region (526–528.9 eV) when cycling between the pristine and the charged to 4.3 V states. Surprisingly, the two peaks at 529.5 and 532 eV, which are also connected to the Mn–O and Ni–O hybridization, decrease in intensity upon charging to 4.3 V, and instead, an onset shoulder extending down to 528.5 eV develops.

Additionally, there is a peak at 534.8 eV that emerges for the Dis 2.2 V sample, which probably originates from a layer of carbonate that forms on discharge in the initial cycles of battery operation as a cathode-electrolyte interphase (CEI).^37^ Furthermore, the presence of a sufficiently thick sodium carbonate film on top of the cathode particles would explain the suppression of intensity in the O K-edge XAS between 526 to 532.8 eV observed for the Dis 2.2 V sample.

RIXS spectra shown in Figure 11d reveal enhancements in several energy loss regions, e.g., between −5.2 to −3.8 eV and −8.0 to −6.4 eV, that are typical for oxygen redox processes that may produce trapped oxygen molecules as previously observed for other battery cathode materials.^38^ The intensity increase in the energy loss regions occurs during charge and diminishes mostly reversibly upon discharge. Interestingly, the intensity sharply increases from the pristine to the Ch 3.8 V sample, remains at the same intensity for the Ch 4.3 V sample, and then decreases rapidly in the Dis 3.8 V sample (shown as an enlarged inset). The asymmetry in behavior of the two Ch 3.8 V and Dis 3.8 V materials is tied to the redox of the lattice oxygen.

## 4 Conclusion

Zn-substituted P2/P3–Na_0.75_Mn_0.68_Ni_0.25_Zn_0.07_O_2_ has been synthesized and characterized using a range of techniques. In order to understand the possible synergistic effects of the formation of the P2/P3 composite structure, single-phase Zn–P2 and Zn–P3 were also synthesized. Furthermore, comparison with unsubstituted materials NMN-P3, NMN-P2, and NMN-P2/P3 has permitted understanding the effects of Zn substitution on these materials. From laboratory PXRD, SPXRD, HRTEM, and NPD, superlattices derived from Na^+^/vacancy and TM layer ordering were observed in Zn-substituted materials. With the application of *ex-situ* PXRD, *ex-situ* Raman, and *operando* SPXRD, it is shown that Zn-substituted structures exhibit excellent structural stability with highly reversible structural changes and suppressed phase transformation upon cycling compared with corresponding unsubstituted phases. In addition, composite P2/P3 phases showed enhanced stability and cycling performance in comparison with equivalent single phases. Structural analysis indicates that this improvement is probably driven by reduced structural changes in the composite materials driven by the intergrowth structure. From cyclic voltammetry, XAS, and RIXS analysis, it is demonstrated that Zn-substituted materials displayed enhanced and stabilized Ni redox and also suppressed irreversible anionic redox upon sodiation/desodiation. The target material in this research, Zn–P2/P3, exhibited excellent electrochemical performance with impressive cycling stability (109 mAh g^–1^ and 90% capacity retention after 100 cycles, with an initial capacity of 121 mAh g^–1^), and superior rate capability (99 mAh g^–1^ at a current rate of 500 mA g^–1^). Excellent cycling stability was observed in full cells vs commercial hard carbon. This work demonstrates the potential of using composites of P2/P3 structures, which show well-matched lattice parameters, to produce high-performance positive electrodes for sodium-ion batteries.

## Supplementary Material



## Notes

Profile refinement and refined parameters for pristine and *ex situ* materials from PXRD data; profile fit for pristine P2 material from synchrotron XRD data; refined parameters for pristine P2/P3 material from powder neutron diffraction data; STEM images and SAED patterns; additional electrochemical data (including from full cell, EIS, etc.); *ex situ* Raman spectra (PDF)
