# Supplementary material for: Enhanced Performance by Zn-Substitution in Biphasic P2/P3–Na0.75Mn0.68Ni0.25Zn0.07O2
Source: Chem Mater. 2026 Jun 22;38(13):6845–56. doi: 10.1021/acs.chemmater.6c01041 (PMC13374014; doi:10.1021/acs.chemmater.6c01041)
Supplement: Supplementary file 1 [file cm6c01041_si_001.pdf]

Enhanced performance by Zn-substitution in biphasic P2/P3-$Na_{0.75}Mn_{0.68}Ni_{0.25}Zn_{0.07}O_2$

Yingling Liao,[†◊] *Rachel Gordon,*[†] *Oxana V. Magdysyuk,*[†] *Aaron B. Naden,*[†] *Maximillian G. Stanzione,*[†] *Pontus Tornblom,*[‡] *Moritz Hirsbrunner,*[‡] *Laurent C. Duda,*[‡] *A. Robert Armstrong*[†◊] *

†EaStCHEM, School of Chemistry, University of St Andrews, St Andrews, Fife KY16 9ST, United Kingdom

◊ The Faraday Institution, Quad One, Harwell Science and Innovation Campus, Didcot, OX11 0RA, United Kingdom

‡ Department of Physics and Astronomy, Division of Molecular and Condensed Matter Physics Uppsala University Uppsala, S-75120, Sweden

* ara@st-andrews.ac.uk

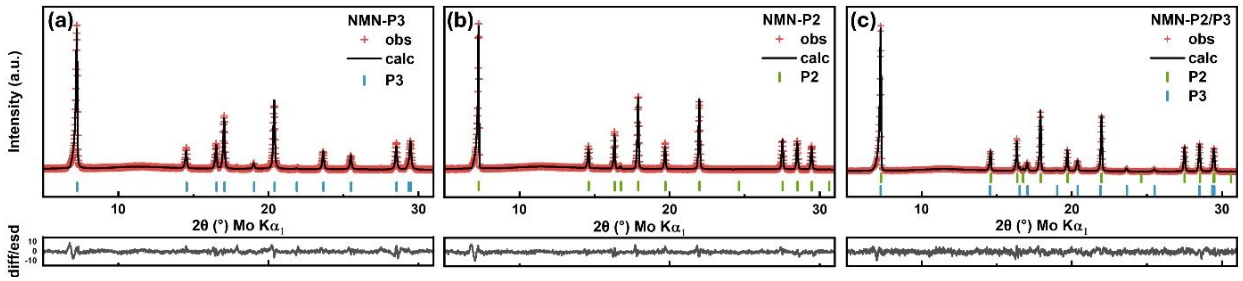

Figure S1. Laboratory X-ray profile fit of as-synthesized unsubstituted materials (a) NMN-P3, (b) NMN-P2 and (c) NMN-P2/P3. Observed data points are shown in red, with fitted profile in black. Green and blue tick marks indicate allowed reflections for the P2 and P3 phases, respectively.

Table S1 Atomic coordinates and isotropic thermal parameters (Biso, $Å^2$) of P3-$Na_{0.7}Mn_{0.75}Ni_{0.25}O_2$ (NMN-P3) pristine obtained from Rietveld refinement shown in **Error! Reference source not found.** (a).

NMN-P3

R_{wp}: 7.09%, R_{exp}: 5.63%, R_p: 5.26%

Lattice parameters P3 Space group *R*3*m* *a* = 2.8789(3) Å, *c* = 16.813(3) Å, V = 120.67 (4) $Å^3$

Atom	Wyckoff symbol	*x*/*a*	*y*/*b*	*z*/*c*	Occupancy	Biso/ $Å^2$
Mn1/Ni1	3a	0	0	0	0.75/0.25	0.24(4)
Na1	3a	0	0	0.1679(6)	0.66(1)	4.2(3)
O1	3a	0	0	0.3902(8)	1	0.32(8)
O2	3a	0	0	0.6016(8)	1	0.32(8)

Table S2 Atomic coordinates and isotropic thermal parameters (Biso, Å^2) of P3-$Na_{0.7}Mn_{0.68}Ni_{0.25}Zn_{0.07}O_2$ (Zn-P3) pristine obtained from Rietveld refinement shown in **Error! Reference source not found.** (d).

Zn-P3						
R_{wp}: 8.34%, R_p: 6.13%, R_e: 6.38% contains 1.1% NiO						
Lattice parameters P3 Space group *R*3*m* *a* = 2.8888(8) Å, *c* = 16.787(7) Å, V = 121.32(8) Å^3						
Atom	Wyckoff symbol	*x*/*a*	*y*/*b*	*z*/*c*	Occupancy	Biso/ Å^2
Mn1/Ni1/Zn1	3a	0	0	0	0.68/0.25/0/07	0.5(3)
Na1	3a	0	0	0.1676(7)	0.702(11)	5.4(4)
O1	3a	0	0	0.3917(10)	1	0.8(2)
O2	3a	0	0	0.6018(9)	1	0.8(2)

Table S3 Atomic coordinates and isotropic thermal parameters (Biso, Å^2) of P2-$Na_{0.7}Mn_{0.75}Ni_{0.25}O_2$ (NMN-P2) pristine obtained from Rietveld refinement shown in **Error! Reference source not found.** (b).

NMN-P2						
R_e: 5.34% R_{wp}: 5.60%, R_p: 4.18%						
Lattice parameters P2 Space group $P6_3/mmc$ *a* = 2.8801(1) Å, *c* = 11.1621(10) Å, V = 80.18 (1) Å^3						
Atom	Wyckoff symbol	*x*/*a*	*y*/*b*	*z*/*c*	Occupancy	Biso/ Å^2
Mn1/Ni1	2*a*	0	0	0	0.75/0.25	0.53(2)
Na1	2*c*	0	0	1/4	0.196(9)	2.2(4)
Na2	2*b*	1/3	2/3	3/4	0.48(1)	6.0(4)
O1	4*f*	1/3	2/3	0.0901(4)	1	0.54(6)

Table S4 Atomic coordinates and isotropic thermal parameters (Biso, Å^2) of P2-$Na_{0.7}Mn_{0.68}Ni_{0.25}Zn_{0.07}O_2$ (Zn-P2) pristine obtained from Rietveld refinement shown in **Error! Reference source not found.** (e).

Zn-P2						
R_{wp}: 9.11%, R_p: 5.99%, R_e: 6.38%<br>Lattice parameters P2 Space group *P*6$_3$/*mmc* *a* = 2.8911(5) Å, *c* = 11.1557(36) Å, V = 80.75 (4) Å^3						
Atom	Wyckoff symbol	*x*/*a*	*y*/*b*	*z*/*c*	Occupancy	Biso/ Å^2
Mn1/Ni1/Zn1	2*a*	0	0	0	0.68/0.25/0.07	0.58(3)
Na1	2*c*	0	0	1/4	0.31(1)	2.3(4)
Na2	2*b*	2/3	1/3	1/4	0.42(1)	5.9(4)
O1	4*f*	1/3	2/3	0.0938(1)	1	0.68(6)

Table S5 Atomic coordinates and isotropic thermal parameters (Biso, Å^2) of P2/P3-$Na_{0.7}Mn_{0.75}Ni_{0.25}O_2$ (NMN-P2/P3) pristine obtained from Rietveld refinement shown in **Error! Reference source not found.** (c).

NMN-P2/P3						
R_{wp}: 6.07%, R_{exp}: 5.27%, R_p: 4.40% Phase ratio: 87% P2, 13% P3<br>Lattice parameters P2 Space group *P*6$_3$/*mmc* *a* = 2.8795(3) Å, *c* = 11.164(2) Å, V = 80.16 (2) Å^3						
Atom	Wyckoff symbol	*x*/*a*	*y*/*b*	*z*/*c*	Occupancy	Biso/ Å^2
Mn1/Ni1	2*a*	0	0	0	0.75/0.25	0.53(3)
Na1	2b	0	0	1/4	0.20(1)	4.2(7)
Na2	2d	2/3	1/3	3/4	0.47(1)	6.3(5)
O1	4*f*	1/3	2/3	0.0911(5)	1	1.28(8)
Lattice parameters P3 Space group *R*3*m* *a* = 2.8792(3) Å, *c* = 16.816(3) Å, V = 120.73(3) Å^3						
Atom	Wyckoff symbol	*x*/*a*	*y*/*b*	*z*/*c*	Occupancy	Biso/ Å^2
Mn1/Ni1	3a	0	0	0	0.75/0.25	0.5
Na1	3a	0	0	0.165(2)	0.63(4)	4
O1	3a	0	0	0.387(2)	1	1
O2	3a	0	0	0.596(2)	1	1

Table S6 Atomic coordinates and isotropic thermal parameters (Biso, Å^2) of P2/P3-$Na_{0.7}Mn_{0.68}Ni_{0.25}Zn_{0.07}O_2$ (Zn-P2/P3) pristine obtained from Rietveld refinement shown in **Error! Reference source not found.** (f).

**Zn-P2/P3**						
R_{wp}: 9.51%, R_p: 6.69, R_e: 8.50%% Phase ratio: 87.0% P2, 13.0% P3						
Lattice parameters P2 Space group $P6_3/mmc$ a = 2.8934(4) Å, c = 11.149(3) Å, V = 80.83 (3) Å^3						
Atom	Wyckoff symbol	x/a	y/b	z/c	Occupancy	Biso/ Å^2
Mn1/Ni1/Zn1	2*a*	0	0	1/2	0.68/0.25/0.07	0.73(4)
Na1	2*c*	0	0	1/4	0.27 (2)	5.0(7)
Na2	2*b*	2/3	1/3	1/4	0.50(2)	5.4(5)
O1	4*f*	1/3	2/3	0.0923(7)	1	1.2(1)
Lattice parameters P3 Space group $R3m$ a = 2.8894(1) Å, c = 16.788 (11) Å, V = 121.38 (12) Å^3						
Atom	Wyckoff symbol	x/a	y/b	z/c	Occupancy	Biso/ Å^2
Mn1/Ni1/Zn1	3a	0	0	0	0.68/0.25/0/07	0.2(1)
Na1	3a	0	0	0.175(2)	0.74(8)	5(2)
O1	3a	0	0	0.392(4)	1	1.1(3)
O2	3a	0	0	0.599(4)	1	1.1(3)

Table S7 Atomic coordinates and isotropic thermal parameters (Biso, Å^2) of 820-P2/P3-$Na_{0.75}Mn_{0.68}Ni_{0.25}Zn_{0.07}O_2$ (820-P2/P3-Zn).

**820-P2/P3-Zn**						
R_{wp}: 7.35%, R_p: 5.46%, R_e: 6.36% Phase ratio: 41.7% P2, 56.3% P3, 2% NiO						
Lattice parameters P2 Space group $P6_3/mmc$, a = 2.8865(2) Å, c = 11.1779(13) Å, V = 80.652 (14) Å^3						
Atom	Wyckoff symbol	*x*/*a*	*y*/*b*	*z*/*c*	Occupancy	Biso/ Å^2
Mn1/Ni1/Zn1	2*a*	0	0	1/2	0.68/0.25/0.07	0.81(7)
Na1	2*c*	0	0	1/4	0.13 (2)	2.2(17)
Na2	2*b*	2/3	1/3	1/4	0.49(3)	8(2)
O1	4*f*	1/3	2/3	0.0854(14)	1	5.5(10)
Lattice parameters P3 Space group $R3m$, a = 2.8878(8) Å, c = 16.796 (9) Å, V = 121.30 (9) Å^3						
Atom	Wyckoff symbol	*x*/*a*	*y*/*b*	*z*/*c*	Occupancy	Biso/ Å^2
Mn1/Ni1/Zn1	3a	0	0	0	0.68/0.25/0/07	0.1(1)
Na1	3a	0	0	0.1705(7)	0.83(4)	7.6(9)
O1	3a	0	0	0.3895(9)	1	0.3(2)
O2	3a	0	0	0.5976(9)	1	0.3(2)

Table S8 Atomic coordinates and isotropic thermal parameters (Biso, Å^2) of 840-P2/P3-$Na_{0.75}Mn_{0.68}Ni_{0.25}Zn_{0.07}O_2$ (840-P2/P3-Zn).

**840-P2/P3-Zn**						
R_{wp}: 9.18%, R_p: 6.54%, R_e: 9.13% Phase ratio: 56.5% P2, 43.5% P3						
Lattice parameters P2 Space group *P*6$_3$/*mmc*, *a* = 2.8924(3) Å, *c* = 11.1658(18) Å, V = 80.90 (2) Å^3						
Atom	Wyckoff symbol	*x*/*a*	*y*/*b*	*z*/*c*	Occupancy	Biso/ Å^2
Mn1/Ni1/Zn1	2*a*	0	0	1/2	0.68/0.25/0.07	1.5(2)
Na1	2*c*	0	0	1/4	0.17(3)	3.3(13)
Na2	2*b*	2/3	1/3	1/4	0.49(3)	6.6(9)
O1	4*f*	1/3	2/3	0.0856(13)	1	2.0(2)
Lattice parameters P3 Space group *R*3*m*, *a* = 2.8906(9) Å, *c* = 16.790 (11) Å, V = 121.50 (11) Å^3						
Atom	Wyckoff symbol	*x*/*a*	*y*/*b*	*z*/*c*	Occupancy	Biso/ Å^2
Mn1/Ni1/Zn1	3a	0	0	0	0.68/0.25/0/07	0.5(2)
Na1	3a	0	0	0.1719(10)	0.89(6)	8.6(13)
O1	3a	0	0	0.3897(14	1	0.6(3)
O2	3a	0	0	0.5971(14)	1	0.6(3)

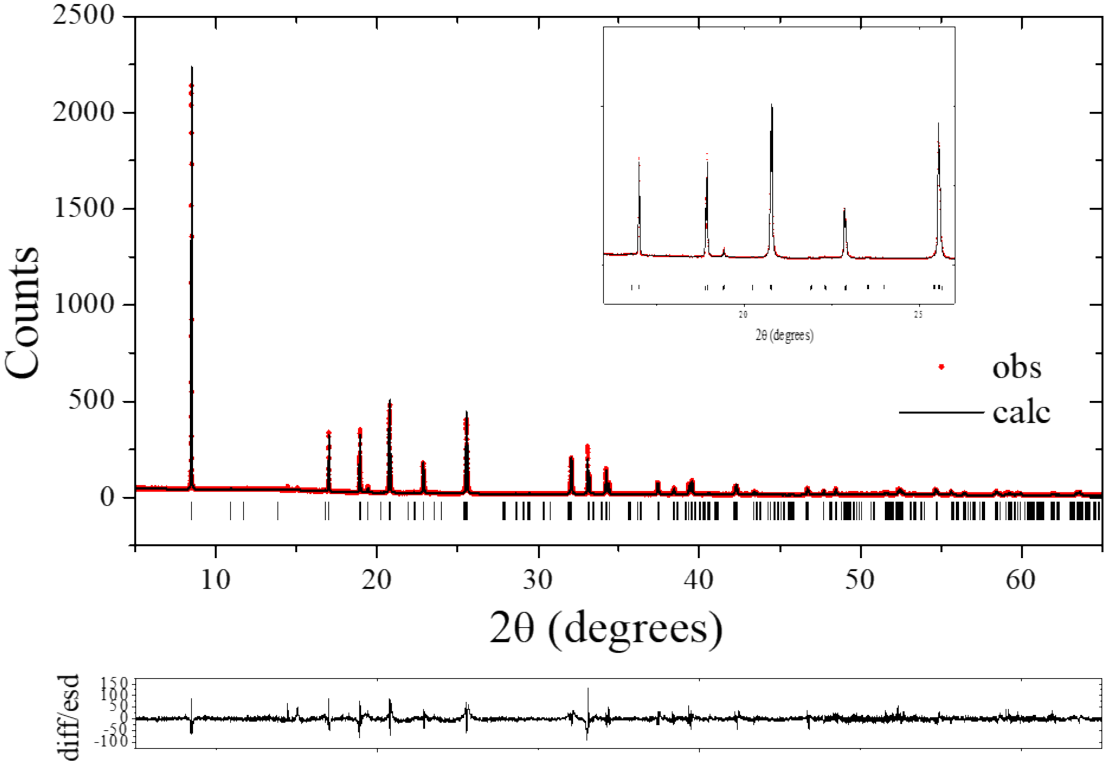

Figure S2. Synchrotron X-ray profile fit of as-synthesized P2-$Na_{0.75}Mn_{0.68}Ni_{0.25}Zn_{0.07}O_2$ in space group *C222₁*. Observed data points are shown in red, with fitted profile in black. Tick marks indicate allowed reflections. Inset shows region between 16 and 27° 2θ to highlight peak splitting.

Table S9 Powder neutron diffraction Rietveld refinement results of P2/P3-$Na_{0.75}Mn_{0.68}Ni_{0.25}Zn_{0.07}O_2$.

**P2/P3-$Na_{0.75}Mn_{0.68}Ni_{0.25}Zn_{0.07}O_2$**						
R_{wp}: 3.93%, R_{exp}: 1.22%, R_p: 4.14% Phase ratio: 89% P2, 8% P3, 3% Na_2CO_3<br>Lattice parameters P2 phase, space group *C222₁* *a* = 5.0135(2) Å, *b* = 8.6779(6), *c* = 11.1515(4) Å						
Atom	Wyckoff symbol	*x*/*a*	*y*/*b*	*z*/*c*	Occupancy	Biso/ Å^2
Mn1/Ni1/Mg1	4b	0	0	1/4	0.818/0.132(6)/0.05	0.3
Mn2/Ni2/Zn2	4b	0	1/3	1/4	0.268/0.572(4)/0.16	0.3
Mn3	4b	0	2/3	3/4	1	0.3
Na1	4a	0	0	0	0.18(4)	2.5
Na2	8c	0	1/3	0.996(4)	0.22(3)	2.5
Na3	4a	0.383(7)	0	0	0.45(6)	2.5
Na4	8c	0.1918(12)	0.1918	0.5	0.36(4)	2.5
O1	8c	0.1721(10)	0.4837(11)	0.8413(7)	1	0.5
O2	8c	0.8235(8)	0.1468(10)	0.3411(5)	1	0.5
O3	8c	0.1477(7)	0.1613(9)	0.8435(6)	1	0.5
Lattice parameters P3 Space group *R3m* *a* = 2.8905 (5) Å, *c* = 16.760(4) Å						

Table S10 The a and c lattice parameters and slab thickness of NMN-P3, NMN-P2, NMN-P2/P3, Zn-P3, Zn-P2, and Zn-P2/P3.

**Composition**	**Shorthand notation**	**Phase**	**lattice parameter (Å)**		**Slab thickness (Å)**
			**a**	**c**	
$Na_{0.7}Mn_{0.75}Ni_{0.25}O_2$	NMN-P3	P3	2.8789(3)	16.813(3)	5.604
	NMN-P2	P2	2.8801(1)	11.1621(10)	5.581
	NMN-P2/P3	P3	2.8792(3)	16.816(3)	5.605
		P2	2.8795(3)	11.164(2)	5.580
$Na_{0.75}Mn_{0.68}Ni_{0.25}Zn_{0.07}O_2$	Zn-P3	P3	2.8888(8)	16.787(7)	5.596
	Zn-P2	P2	2.8911(5)	11.1560(4)	5.578
	Zn-P2/P3	P3	2.8894(1)	16.788 (11)	5.596
		P2	2.8934(4)	11.149(3)	5.575

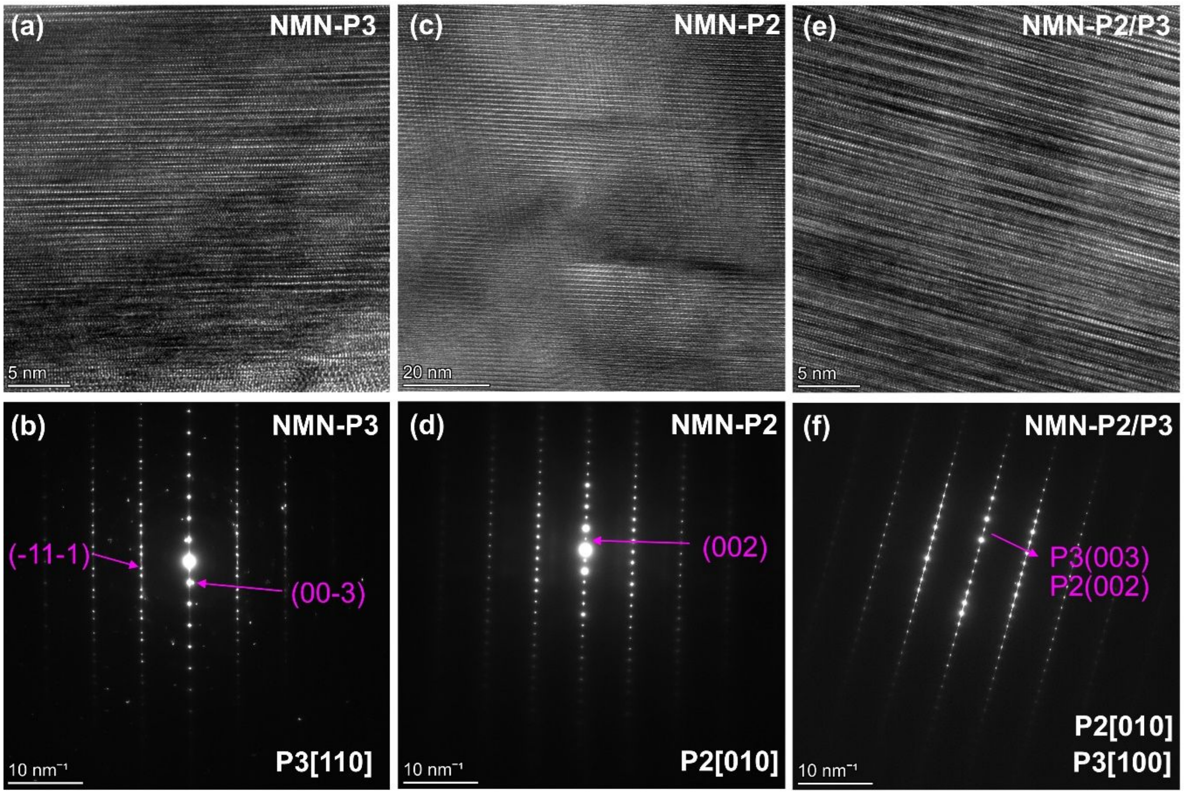

Figure S3. STEM images and corresponding selected area diffraction (SAED) patterns of (a-b) P3-NMN, (c-d) NMN-P2, (e-f) NMN-P2/P3.

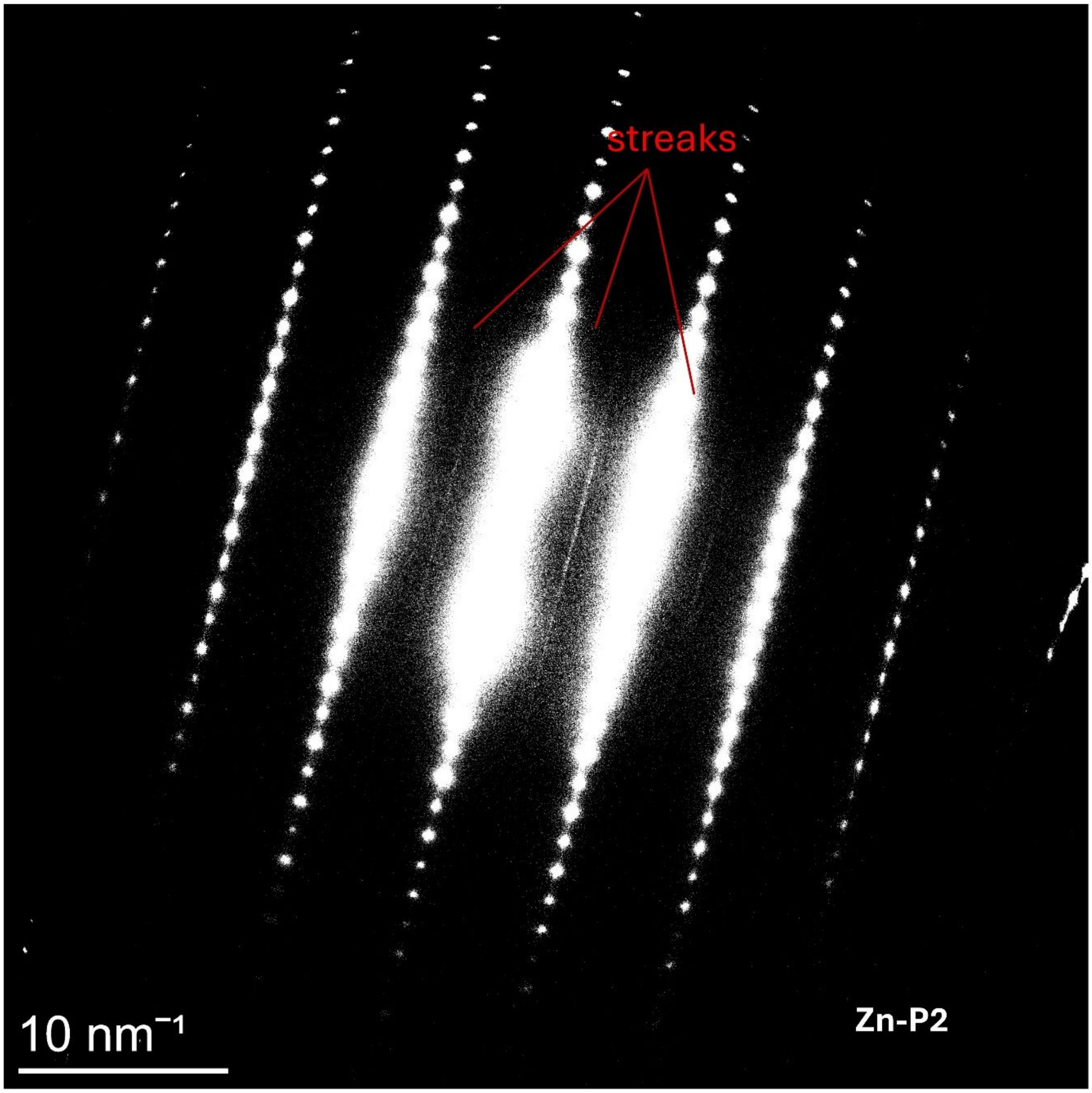

Figure S4 SAED patterns of Zn-P2 after adjusting brightness/contrast of **Error! Reference source not found.** (d).

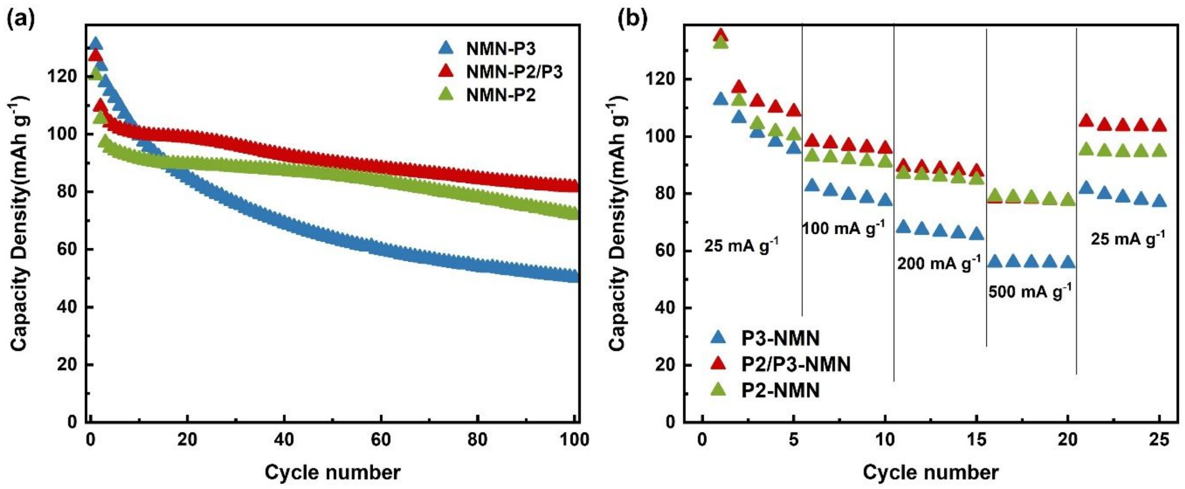

Figure S5(a) Galvanostatic cycling performance, (b) rate performance at 25, 100, 200, 500, and 25 mA g^{-1} of NMN-P3, NMN-P2, and NMN-P2/P3.

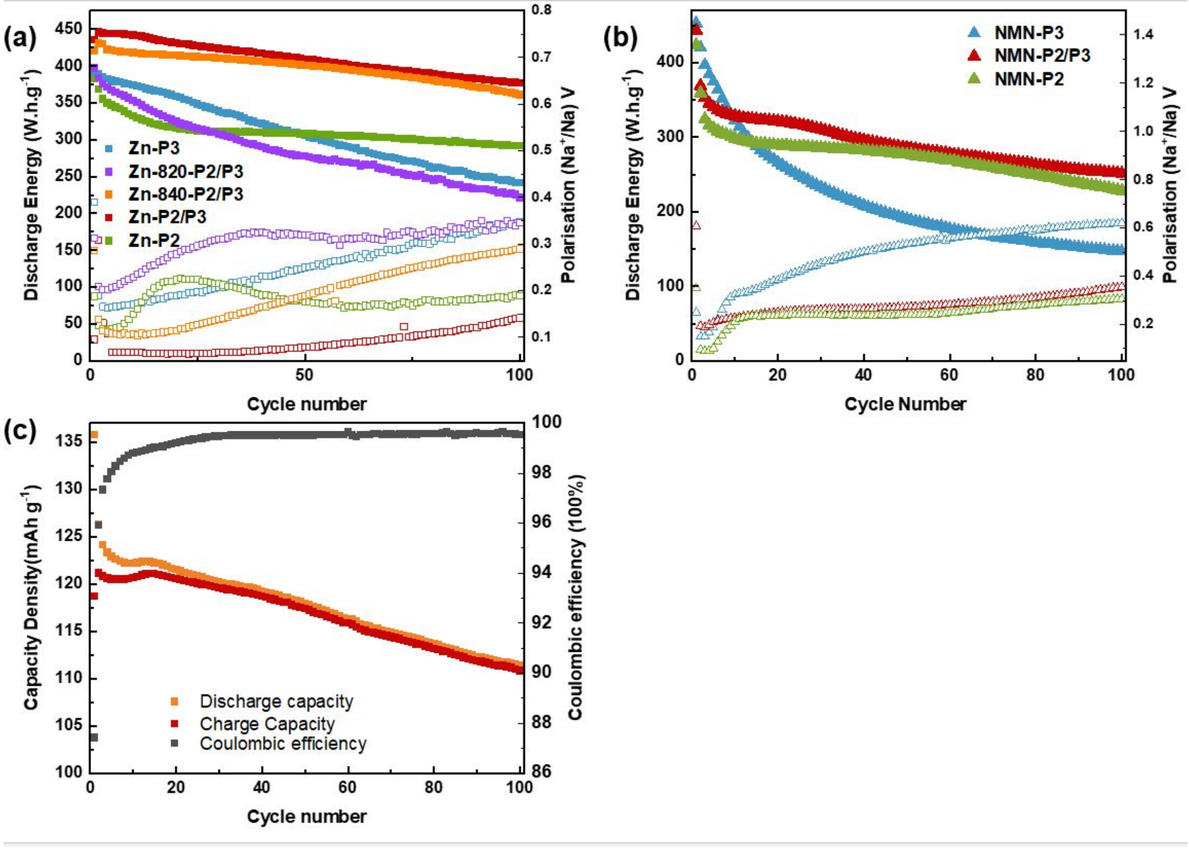

Figure S6 (a) specific discharge energy and polarisation of Zn subsituted samples Zn-P3, Zn-820-P2/P3, Zn-840-P2/P3, Zn-P2/P3 and Zn-P2, (b) unsubstituted NMN-P3, NMN-P2/P3, and NMN-P2. (c) cycling data for Zn-P2/P3 showing Coulombic efficiency.

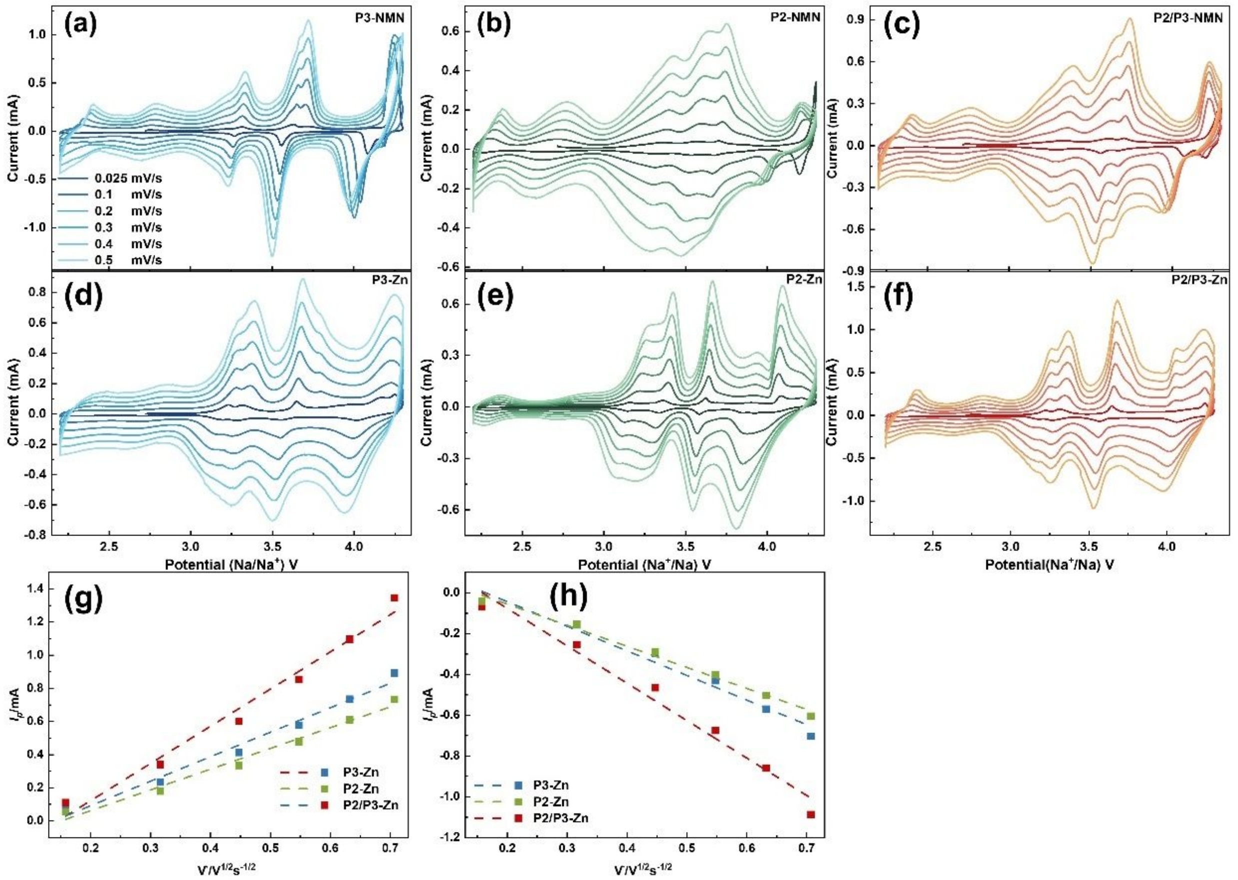

Figure S7. Cyclic voltammograms of (a-f) NMN-P3, NMN-P2/P3, NMN-P2, Zn-P3, Zn-P2/P3, and Zn-P2 between 2.2-4.3 V vs. Na^+/Na with different scan rates: 0.025, 0.1, 0.2, 0.3, 0.4, and 0.5 mV s^{-1}. (g-h) Plots showing relationship between the peak current (i_p) and the square root of the scan rate ($\nu^{1/2}$).

(a)

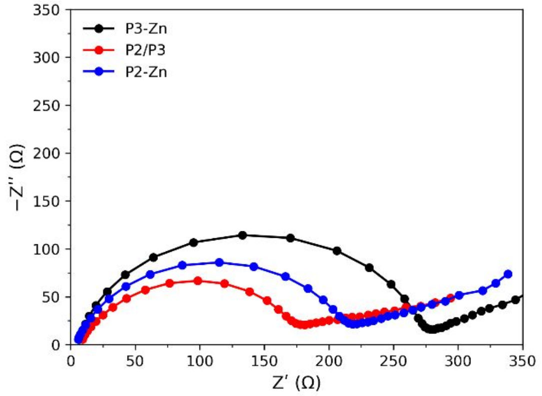
P3-Zn
P2/P3
P2-Zn
−Z'' (Ω)
Z' (Ω)

(b)

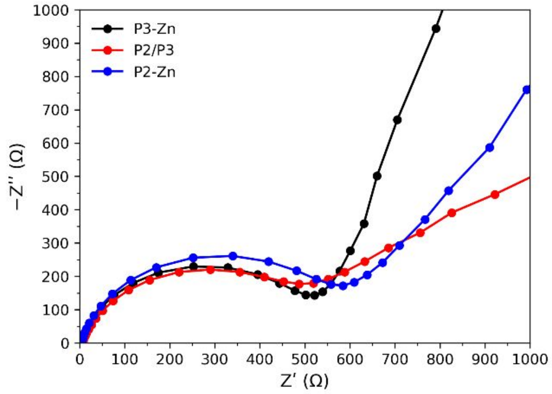
P3-Zn
P2/P3
P2-Zn
−Z'' (Ω)
Z' (Ω)

(c)

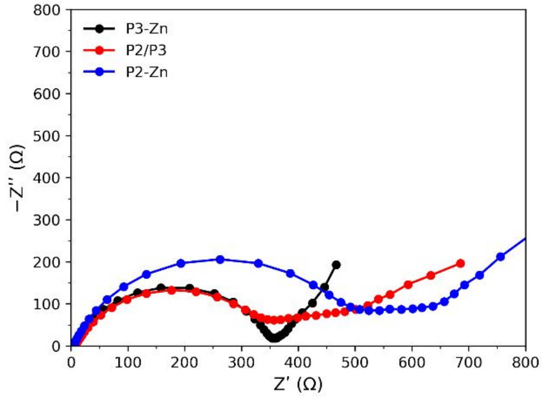
P3-Zn
P2/P3
P2-Zn
−Z'' (Ω)
Z' (Ω)

(d)

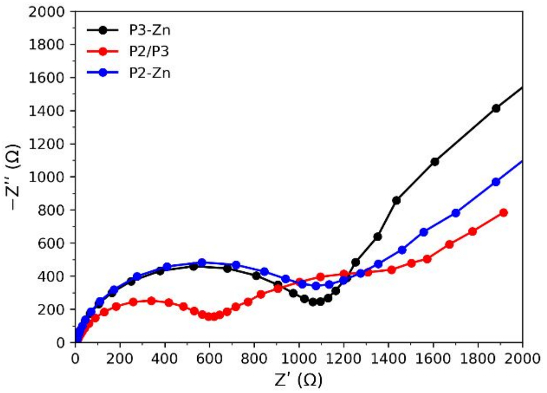
P3-Zn
P2/P3
P2-Zn
−Z'' (Ω)
Z' (Ω)

(e)

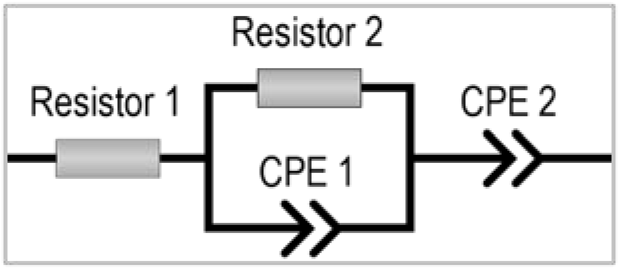
Resistor 2
Resistor 1
CPE 1
CPE 2

(f)

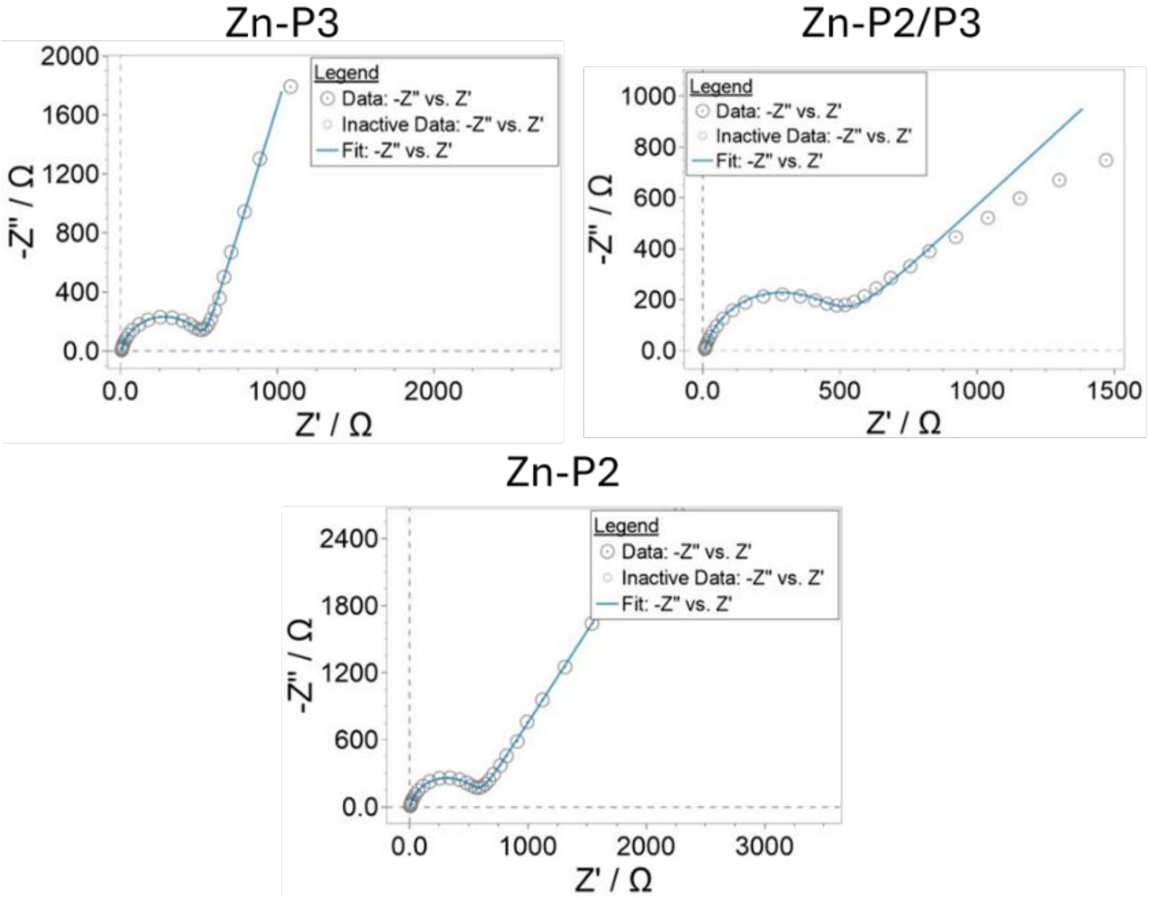

Figure S8. Electrochemical impedance spectroscopy (EIS) data for Zn-P3, Zn-P2/P3 and Zn-P2 at (a) pristine state, (b) charged to 3.5 V, (c) charged to 4.3 V (d) discharged to 2.2 V. (e) equivalent circuit model used to fit the EIS data (f) The corresponding fits of the pristine samples using the equivalent circuit model in (e).

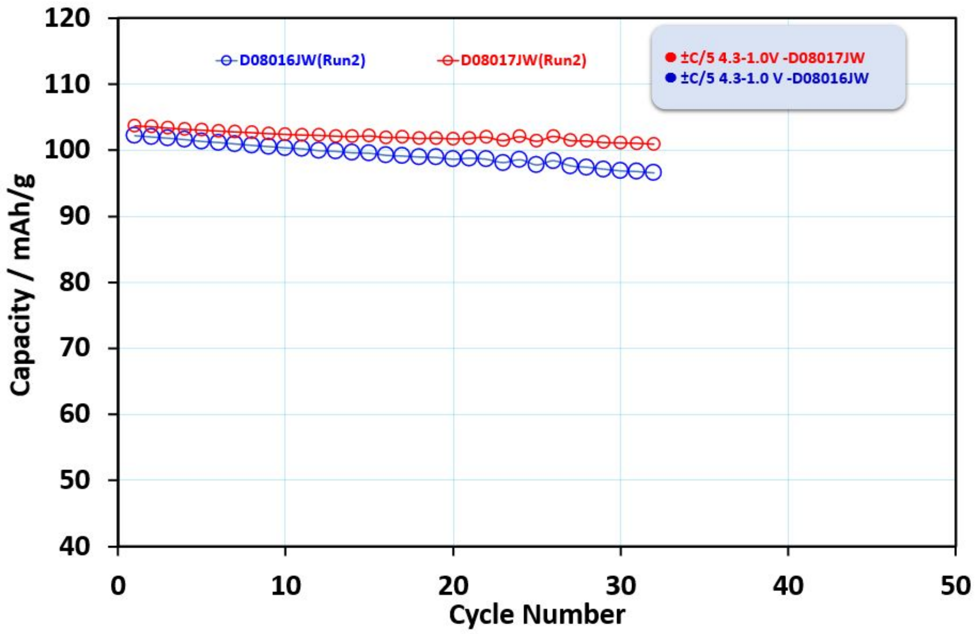

Figure S9. Cycling data from 3-electrode pouch cells for Zn-P2/P3 in the voltage window 1-4.3V at C/5.

Figure S10(a) illustrates the two Raman active modes for each transition metal in the unit cell, E_g (doubly degenerate symmetric to inversion centre) mode is an opposite bending motion of oxygen along adjacent O-layers, which is assigned to a lower wavenumber (around 478 cm^{-1}). Whereas, in A_{1g} (singly degenerate symmetric to σ_v or perpendicular C_v, and symmetric to inversion centre) mode oxygens move symmetrically along the *c*-axis and stretch, and assigned to a higher wavenumber (around 587 cm^{-1}).[1]

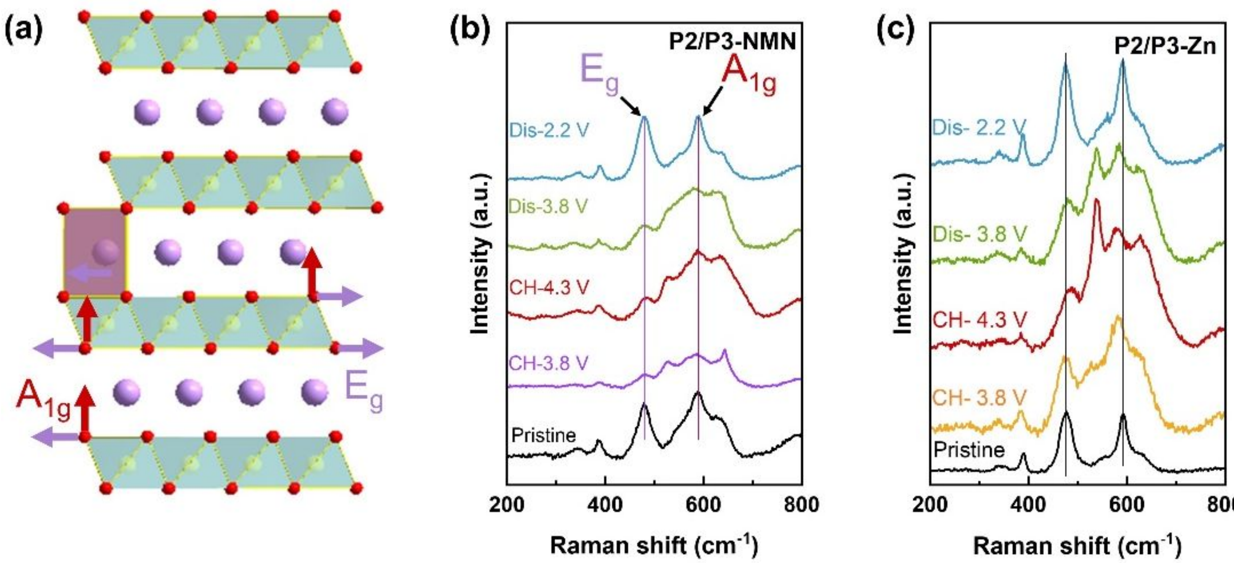

Figure S10(a) Schematic representation of the $NaTMO_2$ unit cell. The atomic displacements from E_g and A_{1g} vibrational modes are shown in purple and red, respectively. Ex-situ Raman spectra of (b) NMN-P2/P3 and (c) Zn-P2/P3. Ex-situ materials were collected for pristine, the first charge to 3.8 V, the first charge to 4.3 V, the first discharge to 3.8 V, and the first discharge to 2.2 V.

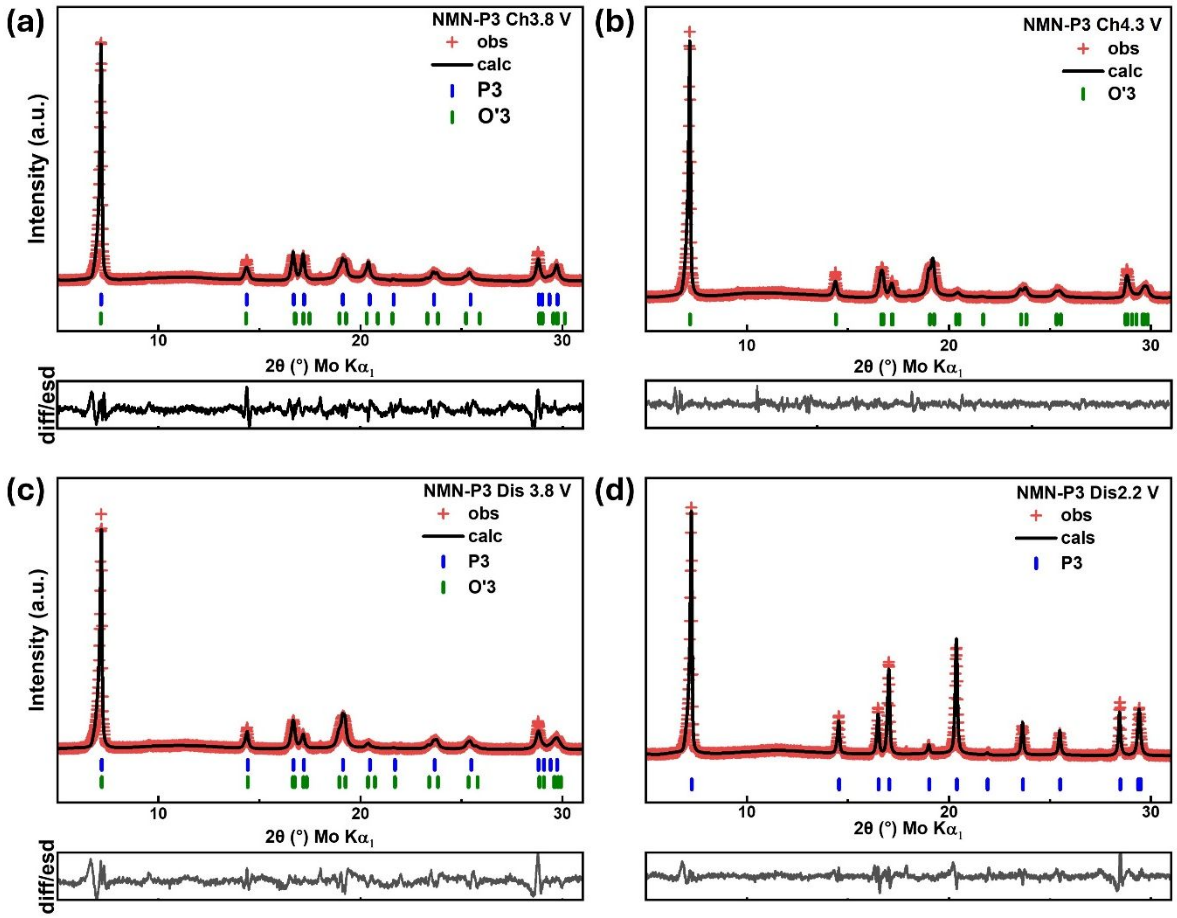

Figure S11 Laboratory X-ray Rietveld fits of NMN-P3 (a) charged to 3.8 V, (b) charged to 4.3 V, (c) discharged to 3.8 V after charge to 4.3 V and (d) discharge to 2.2 V after charge to 4.3 V. Observed data points are shown in red, with fitted profile in black. Blue and green tick marks indicate P2 and O’3 allowed reflections, respectively.

Table S11 Atomic coordinates and isotropic thermal parameters (Biso/Å^2) of P3-$Na_{0.7}Mn_{0.75}Ni_{0.25}O_2$ (P3-NMN) charged to 3.8 V from the Rietveld refinement shown in Figure S (a).

P3-NMN charged to 3.8 V						
R_e: 5.25%, R_{wp}: 7.13%, R_{exp}:5.25%, R_p: 5.31%, Phase ratio: 47.0% P3, 53.0% O'3						
Lattice parameters P3 Space group *R*3*m* *a* = 2.8563(4) Å, *c* = 17.023(3) Å, V = 120.28(4) Å^3						
Atom	Wyckoff symbol	*x*/*a*	*y*/*b*	*z*/*c*	Occupancy	Biso/ Å^2
Mn1/Ni1	3a	0	0	0	0.75/0.25	1.6 (2)
Na1	3a	0	0	0.169(2)	0.55(2)	4
O1	3a	0	0	0.393(2)	1	1.5(3)
O2	3a	0	0	0.600(2)	1	1.5(3)
Lattice parameters O'3 Space group *C2/m, a* = 4.935(2) Å, *b* = 2.8513(8) Å, *c* = 5.826(3) Å, β = 106.29(4)^o, V = 78.68(6) Å^3						
Atom	Wyckoff symbol	*x*/*a*	*y*/*b*	*z*/*c*	Occupancy	Biso/ Å^2
Mn1/Ni1	2a	0	0	0	0.75/0.25	0.6
Na1	2d	0	0.5	0.5	0.25	3
O1	4i	0.675(3)	0	0.182(2)	1	0.7

Table S9 Atomic coordinates and isotropic thermal parameters (Biso/Å^2) of P3-$Na_{0.7}Mn_{0.75}Ni_{0.25}O_2$ (P3-NMN) charged to 4.3 V from the Rietveld refinement shown in Figure S (b).

P3-NMN charged to 4.3 V						
R_{wp}: 7.75%, R_{exp}:5.38%, R_p: 5.73%, 100% O'3						
Lattice parameters O'3 Space group *C*2/*m* *a* = 4.9312(9) Å, *b* = 2.8523(6) Å, *c* = 5.886(2) Å, β = 106.08(2)^o, V = 79.55(4) Å^3						
Atom	Wyckoff symbol	*x*/*a*	*y*/*b*	*z*/*c*	Occupancy	Biso/ Å^2
Mn1/Ni1	2a	0	0	0	0.75/0.25	0.6
Na1	2d	0	0.5	0.5	0.34(2)	3
O1	4i	0.680(2)	0	0.165(3)	1	1.0(2)

Table S10 Atomic coordinates and isotropic thermal parameters (Biso/Å^2) of P3-$Na_{0.7}Mn_{0.75}Ni_{0.25}O_2$ (P3-NMN) discharged to 3.8 V from the Rietveld refinement shown in Figure S (c).

**P3-NMN discharged to 3.8 V**						
R_{wp}: 7.91%, R_{exp}: 5.15%, R_p: 6.09%, Phase ratio: 11.9% O'3, 88.1% P3						
Lattice parameters P3 Space group *R3m* *a* = 2.8485(4) Å, *c* = 16.983(10) Å, V = 119.33 (8) Å^3						
Atom	Wyckoff symbol	*x*/*a*	*y*/*b*	*z*/*c*	Occupancy	Biso/ Å^2
Mn1/Ni1	3a	0	0	0	0.75/0.25	0.5
Na1	3a	0	0	0.14 (1)	0.56 (10)	3.5
O1	3a	0	0	0.38 (1)	1	1
O2	3a	0	0	0.62 (2)	1	1
Lattice parameters O'3 Space group *C2/m* *a* = 4.941(3) Å, *b* = 2.848(2) Å, *c* = 5.919(3) Å, b = 107.18(4) °, V = 79.58 (8) Å^3						
Atom	Wyckoff symbol	*x*/*a*	*y*/*b*	*z*/*c*	Occupancy	Biso/ Å^2
Mn1/Ni1	2a	0	0	0	0.75/0.25	1.5(2)
Na1	2d	0	0.5	0.5	0.50(3)	7
O1	4i	0.681(2)	0	0.177(2)	1	1

Table S11 Atomic coordinates and isotropic thermal parameters (Biso/Å^2) of P3-$Na_{0.7}Mn_{0.75}Ni_{0.25}O_2$ (P3-NMN) discharged to 3.8 V from the Rietveld refinement shown in Figure S (d).

**P3-NMN discharged to 2.2 V**						
R_e: 5.43%, R_{wp}: 8.09%, R_p: 5.92% 100% P3						
Lattice parameters P3 Space group *R3m* *a* = 2.8837(3) Å, *c* = 16.7948(36) Å, V = 120.95 (4) Å^3						
Atom	Wyckoff symbol	*x*/*a*	*y*/*b*	*z*/*c*	Occupancy	Biso/ Å^2
Mn1/Ni1	3*a*	0	0	0	0.75/0.25	0.38(5)
Na1	3*a*	0	0	0.1668(1)	0.72(1)	6.9(4)
O1	3*a*	0	0	0.3905(10)	1	0.53(10)
O2	3*a*	0	0	0.6003(9)	1	0.53(10)

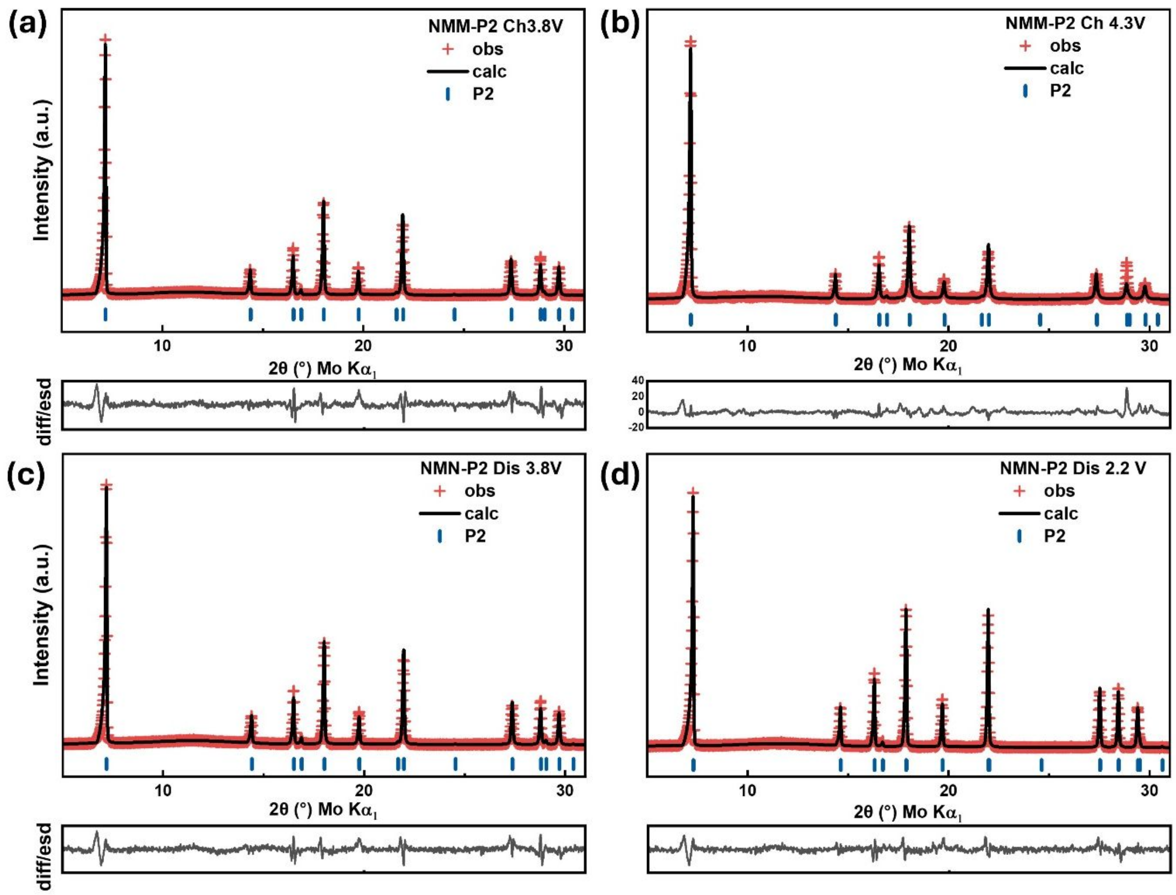

Figure S12 *Laboratory X-ray Rietveld fits of NMN-P2 (a) charged to 3.8 V, (b) charged to 4.3 V, (c) discharged to 3.8 V after charge to 4.3 V and (d) discharge to 2.2 V after charge to 4.3 V. Observed data points are shown in red, with fitted profile in black. Blue and green tick marks indicate P2 and O'3 allowed reflections, respectively.*

Table S12 Atomic coordinates and isotropic thermal parameters (Biso/ Å^2) of P2-$Na_{0.7}Mn_{0.75}Ni_{0.25}O_2$ (NMN-P2) first charge to 3.8 V, obtained from the Rietveld refinement shown in Figure S (a).

NMN-P2 Charged to 3.8 V						
R_e: 5.16%, R_{wp}: 8.06%, R_p: 5.82% 100% P2						
Lattice parameters P2 Space group $P6_3/mmc$ a = 2.8501(5) Å, c = 11.327(4) Å, V = 79.68 (4) Å^3						
Atom	Wyckoff symbol	x/a	y/b	z/c	Occupancy	Biso/ Å^2
Mn1/Ni1	2a	0	0	0	0.75/0.25	0.72(3)
Na1	2c	0	0	1/4	0.068(13)	4.3(1.9)
Na2	2b	1/3	2/3	3/4	0.36(1)	5.9(5)
O1	4f	1/3	2/3	0.0871(4)	1	0.73(7)

Table S13 Atomic coordinates and isotropic thermal parameters (Biso/Å^2) of P2-$Na_{0.7}Mn_{0.75}Ni_{0.25}O_2$ (NMN-P2) first charge to 4.3 V from the Rietveld refinement shown in Figure S (b).

NMN-P2 charged to 4.3 V						
R_{wp}: 12.30%, R_{exp}:5.21%, R_p: 8.97%						
Lattice parameters P2 Space group $P6_3/mmc$ a = 2.8451(5) Å, c = 11.339(4) Å, V = 79.48 (3) Å^3						
Atom	Wyckoff symbol	x/a	y/b	z/c	Occupancy	Biso/ Å^2
Mn1/Ni1	2a	0	0	0	0.75/0.25	1.56(7)
Na1	2c	0	0	1/4	0.096(11)	4
Na2	2b	1/3	2/3	3/4	0.33(3)	4.5(9)
O1	4f	1/3	2/3	0.088(1)	1	2.6 (2)

Table S14 Atomic coordinates and isotropic thermal parameters (Biso/ Å^2) of P2-$Na_{0.7}Mn_{0.75}Ni_{0.25}O_2$ (NMN-P2) first discharge to 3.8 V from the Rietveld refinement shown in Figure S (c).

NMN-P2 discharged to 3.8 V						
R_e: 5.21%, R_{wp}: 6.95%, R_p: 5.17% 100% P2<br>Lattice parameters P2 Space group *P*6_3/*mmc* *a* = 2.8526(1) Å, *c* = 11.314(1) Å, V = 79.73 (1) Å^3						
Atom	Wyckoff symbol	*x*/*a*	*y*/*b*	*z*/*c*	Occupancy	Biso/ Å^2
Mn1/Ni1	2*a*	0	0	0	0.75/0.25	0.56(3)
Na1	2*c*	0	0	1/4	0.14(1)	5.9(11)
Na2	2*b*	1/3	2/3	3/4	0.34(1)	5.4(5)
O1	4*f*	1/3	2/3	0.0883(3)	1	0.47(6)

Table S15 Atomic coordinates and isotropic thermal parameters (Biso/ Å^2) of P2-$Na_{0.7}Mn_{0.75}Ni_{0.25}O_2$ (NMN-P2) first discharge to 2.2 V from Rietveld refinement shown in Figure S (d).

NMN-P2 discharged to 2.2 V						
R_e: 5.33%, R_{wp}: 6.34%, R_p: 4.77% 100% P2<br>Lattice parameters P2 Space group *P*6_3/*mmc* *a* = 2.8863(2) Å, *c* = 11.1392(14) Å, V = 80.362(17) Å^3						
Atom	Wyckoff symbol	*x*/*a*	*y*/*b*	*z*/*c*	Occupancy	Biso/ Å^2
Mn1/Ni1	2*a*	0	0	0	0.75/0.25	0.52(2)
Na1	2*c*	0	0	1/4	0.227(9)	4.4(5)
Na2	2*b*	1/3	2/3	3/4	0.458(10)	4.6(3)
O1	4*f*	1/3	2/3	0.0913(3)	1	0.70(5)

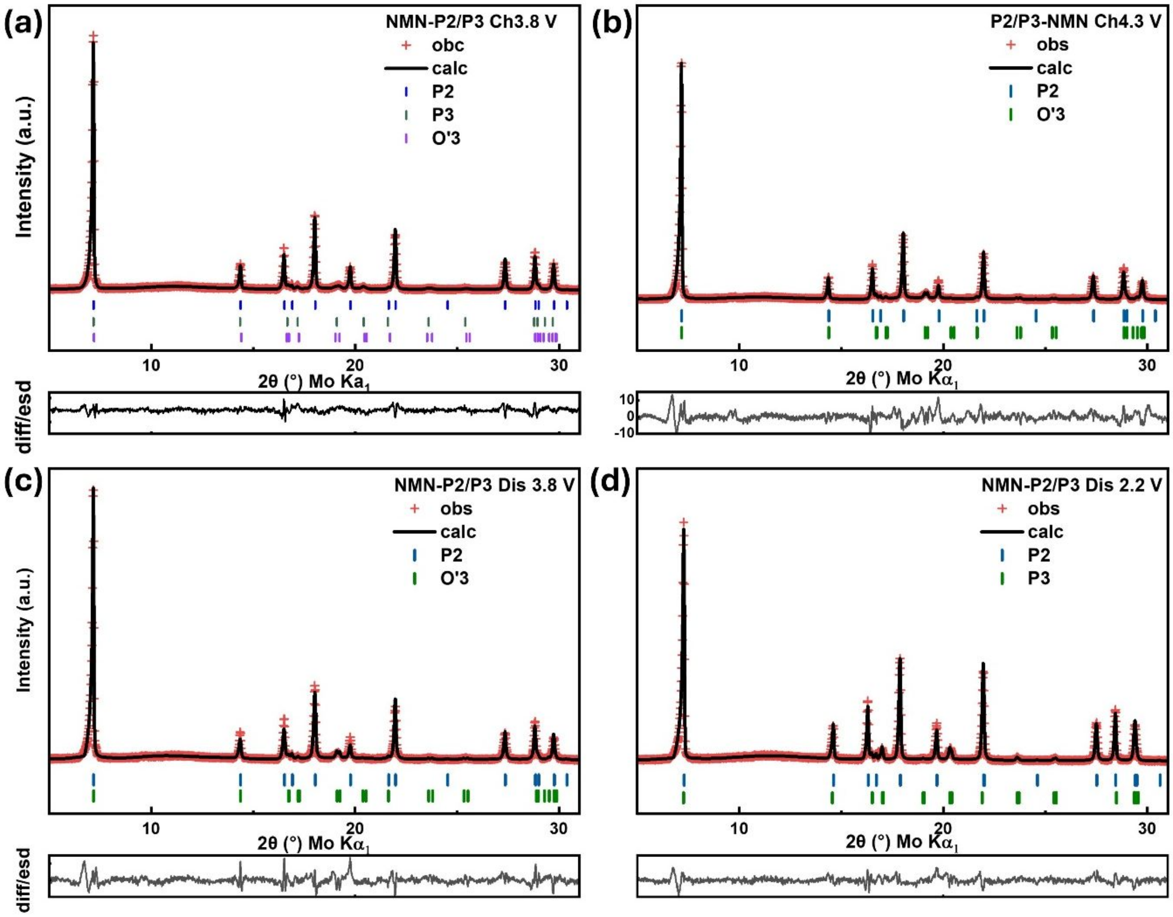

Figure S13 *Laboratory X-ray Rietveld fits of NMN-P2/P3 (a) charged to 3.8 V, (b) charged to 4.3 V, (c) discharged to 3.8 V after charge to 4.3 V and (d) discharge to 2.2 V after charge to 4.3 V. Observed data points are shown in red, with fitted profile in black. Blue and green tick marks indicate P2 and O'3 allowed reflections, respectively.*

Table S16 Atomic coordinates and isotropic thermal parameters (Biso/Å^2) of P2/P3-$Na_{0.7}Mn_{0.75}Ni_{0.25}O_2$ (NMN-P2/P3) charged to 3.8 V obtained from Rietveld refinement shown in Figure S (a).

**NMN-P2/P3 Charged to 3.8 V**						
Rwp: 7.81%, R_{exp}: 5.25%, Rp: 4.94%, Phase ratio: 91% P2, 3% P3, 6% O'3						
Lattice parameters P2 Space group $P6_3/mmc$ a = 2.8499(3) Å, c = 11.332(3) Å, V = 79.70 (3) Å^3						
Atom	Wyckoff symbol	*x*/*a*	*y*/*b*	*z*/*c*	Occupancy	Biso/ Å^2
Mn1/Ni1	2*a*	0	0	0	0.75/0.25	0.79(4)
Na1	2b	0	0	1/4	0.129(7)	6.3(5)
Na2	2d	2/3	1/3	3/4	0.34(1)	6.3(5)
O1	4*f*	1/3	2/3	0.0869(4)	1	0.79(9)
Lattice parameters O'3 Space group C2/m, *a* = 4.952(6) Å, *b* = 2.838(2) Å *c* = 5.900 (5) Å, *β* =106.78 (7), volume = 79.37 (13) Å^3						
Lattice parameters P3 Space group R3m *a* = 2.855(2) Å, *c* = 17.05 (2) Å, volume = 120.4 (2) Å^3						

Table S17 Atomic coordinates and isotropic thermal parameters (Biso/Å^2) of P2/P3-$Na_{0.7}Mn_{0.75}Ni_{0.25}O_2$ (NMN-P2/P3) charge to 4.3 V obtained from Rietveld refinement shown in Figure S (b).

**NMN-P2/P3 Charged to 4.3 V**						
R_{wp}: 7.77%, R_{exp}: 5.05% R_p: 5.72%, Phase ratio: 86. 4% P2, 13.6% O‘3						
Lattice parameters P2 Space group $P6_3/mmc$ a = 2.8469(3) Å, c = 11.338(3) Å, V = 79.58 (2) Å^3						
Atom	Wyckoff symbol	*x*/*a*	*y*/*b*	*z*/*c*	Occupancy	Biso/ Å^2
Mn1/Ni1	2*a*	0	0	0	0.75/0.25	1.55(5)
Na1	2b	0	0	1/4	0.052(10)	4
Na2	2d	2/3	1/3	3/4	0.36(2)	6.6(9)
O1	4*f*	1/3	2/3	0.0847(7)	1	1.3(1)
Lattice parameters O’3 Space group *C2/ma* = 4.942(2) Å, *b* = 2.843(2) Å, *c* = 5.895 (4) Å, β = 105.88 (4)°, V = 79.68 (8) Å^3						
Atom	Wyckoff symbol	*x*/*a*	*y*/*b*	*z*/*c*	Occupancy	Biso/ Å^2
Mn1/Ni1	3a	0	0	0	0.75/0.25	0.6
Na1	3a	0	0	1/2	0.25	3
O1	3a	0.680(2)	0	0.170(7)	1	0.7

Table S18 Atomic coordinates and isotropic thermal parameters (Biso/Å^2) of P2/P3-$Na_{0.7}Mn_{0.75}Ni_{0.25}O_2$ (NMN-P2/P3) discharge to 3.8 V from Rietveld refinement shown in Figure S (c).

**NMN-P2/P3 Discharged to 3.8 V**						
R_{wp}: 7.10%, R_{exp}: 5.28%, R_p: 5.38%, Phase ratio: 89% P2, 11% O'3						
Lattice parameters P2 Space group *P*6$_3$/*mmc* *a* = 2.8484(6) Å, *c* = 11.329(5) Å, V = 79.60 (5) Å^3						
Atom	Wyckoff symbol	*x*/*a*	*y*/*b*	*z*/*c*	Occupancy	Biso/ Å^2
Mn1/Ni1	2*a*	0	0	0	0.75/0.25	1.13(4)
Na1	2b	0	0	1/4	0.11(1)	4
Na2	2d	2/3	1/3	3/4	0.31(1)	3.7(5)
O1	4*f*	1/3	2/3	0.0872(5)	1	1.15(10)
Lattice parameters O'3 Space group *C2/m* *a* = 4.947(3) Å, *b* = 2.848(6) Å, *c* = 5.813 (9) Å, β = 105.41(6)^o, V = 78.94 (20) Å^3						
Atom	Wyckoff symbol	*x*/*a*	*y*/*b*	*z*/*c*	Occupancy	Biso/ Å^2
Mn1/Ni1	3a	0	0	0	0.75/0.25	0.5
Na1	3a	0	0	1/2	0.44(7)	4
O1	3a	0.698(8)	0	0.191(8)	1	1

Table S192 Atomic coordinates and isotropic thermal parameters (Biso/Å^2) of P2/P3-$Na_{0.7}Mn_{0.75}Ni_{0.25}O_2$(NMN-P2/P3) discharge to 2.2 V from Rietveld refinement shown in Figure S (d).

**NMN-P2/P3 Discharged to 2.2 V**						
R_{wp}: 6.05%, R_{exp}: 5.36% R_p: 4.59%, Phase ratio: 92% P2, 8% P3						
Lattice parameters P2 Space group *P*6$_3$/*mmc* *a* = 2.8863(6) Å, *c* = 11.150(4) Å, V = 80.44 (4) Å^3						
Atom	Wyckoff symbol	*x*/*a*	*y*/*b*	*z*/*c*	Occupancy	Biso/ Å^2
Mn1/Ni1	2*a*	0	0	0	0.75/0.25	0.59(3)
Na1	2b	0	0	1/4	0.18(1)	3.0(7)
Na2	2d	2/3	1/3	3/4	0.50(2)	6.4(5)
O1	4*f*	1/3	2/3	0.0904(5)	1	0.63(7)
Lattice parameters P3 Space group *R*3*m* *a* = 2.8843(15) Å, *c* = 16.796(16) Å, V = 121.02 (17) Å^3						
Atom	Wyckoff symbol	*x*/*a*	*y*/*b*	*z*/*c*	Occupancy	Biso/ Å^2
Mn1/Ni1	3a	0	0	0	0.75/0.25	0.5
Na1	3a	0	0	0.151(2)	0.85(5)	3
O1	3a	0	0	0.419(3)	1	0.7
O2	3a	0	0	0.604(3)	1	0.7

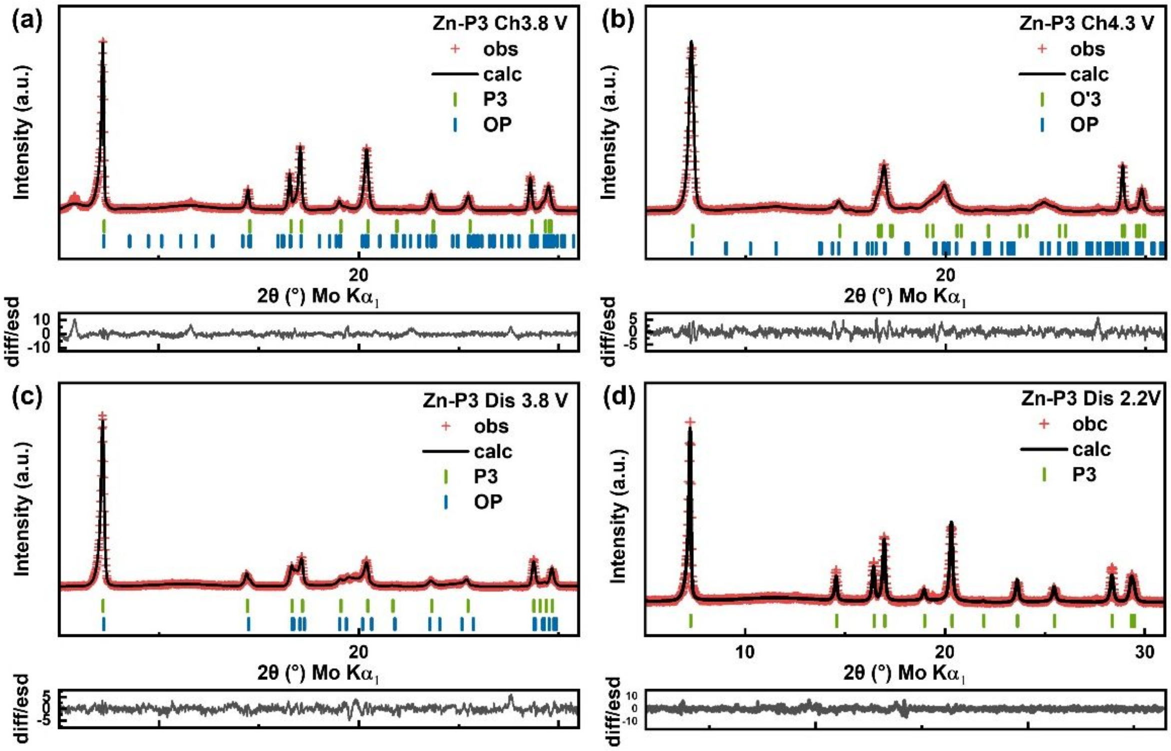

Figure S14 Laboratory X-ray Rietveld fits of Zn-P3 (a) charged to 3.8 V, (b) charged to 4.3 V, (c) discharged to 3.8 V after charge to 4.3 V and (d) discharge to 2.2 V after charge to 4.3 V. Observed data points are shown in red, with fitted profile in black.

Table S20 Atomic coordinates and isotropic thermal parameters (Biso, Å^2) of P3-$Na_{0.75}Mn_{0.68}Ni_{0.25}Zn_{0.07}O_2$ (Zn-P3) charge to 3.8 V obtained from the Rietveld refinement shown in Figure S (a).

**P3 $Na_{0.75}Mn_{0.68}Ni_{0.25}Zn_{0.07}O_2$ charge to 3.8 V**						
R_{wp}: 8.79%, R_{exp}: 6.52%, R_p: 6.10% Phase ratio: 71.14% Na ordered structure, 28.86% P3						
Lattice parameters Na ordered structure Space group *P2₁/m* *a* = 4.976(1) Å, *b* = 5.740(2) Å, *c* = 5.860(2) Å, β = 106.63(4) °, V = 160.38(9) Å^3						
Atom	Wyckoff symbol	*x*/*a*	*y*/*b*	*z*/*c*	Occupancy	Biso/ Å^2
Mn1/Ni1/Zn1	3a	0	0	1/2	0.68/0.25/0/07	0.8
Mn2/Ni2/Zn2	3a	0.500(5)	1/4	0.500(2)	0.68/0.25/0/07	0.8
Na1	3a	0.45(3)	1/4	0.29(2)	0.26(5)	1
Na2	3a	0.158(5)	0	0.011(5)	0.51(2)	1
O1	3a	0.91(1)	1/4	0.683(9)	1	11
O2	3a	0.11(1)	1/4	0.358(9)	1	11
O3	3a	0.581(6)	0.026(6)	0.314(9)	1	1
Lattice parameters P3 Space group *R3m* *a* = 2.866(2) Å, *c* = 16.80 (2) Å, V = 119.51(19) Å^3						
Atom	Wyckoff symbol	*x*/*a*	*y*/*b*	*z*/*c*	Occupancy	Biso/ Å^2
Mn1/Ni1/Zn1	3a	0	0	0	0.68/0.25/0.07	0.5
Na1	3a	0	0	0.218(7)	0.44(9)	1
O1	3a	0	0	0.39(2)	1	1
O2	3a	0	0	0.61(2)	1	1

Table S21 Atomic coordinates and isotropic thermal parameters (Biso, Å^2) of P3-$Na_{0.75}Mn_{0.68}Ni_{0.25}Zn_{0.07}O_2$ (Zn-P3) charge to 4.3 V obtained from the Rietveld refinement shown in Figure S (b).

Zn-P3 charged 4.3 V

R_{wp}: 9.21%, R_{exp}: 6.39%, R_p: 6.99%   Phase ratio: 85.42% O'3, 14.58% P3

Lattice parameters O'3 Space group *C2/m* *a* = 4.907(1) Å, *b* = 2.8522(8) Å, *c* = 5.625(3) Å, β = 108.50(3) °, V = 74.66(5) Å^3

Atom	Wyckoff symbol	*x*/*a*	*y*/*b*	*z*/*c*	Occupancy	Biso/ Å^2
Mn1/Ni1/Zn1	3*a*	0	0	0	0.68/0.25/0.07	0.5
Na1	3*a*	0	0	1/2	0.23(4)	5
O1	3*a*	0.763(4)	0	0.183(4)	1	1

Lattice parameters P3 Space group *R3m* *a* = 2.8288(27) Å, *c* = 16.74 (2) Å, V = 116.00(5) Å^3

Atom	Wyckoff symbol	*x*/*a*	*y*/*b*	*z*/*c*	Occupancy	Biso/ Å^2
Mn1/Ni1/Zn1	3a	0	0	0	0.68/0.25/0.07	0.5
Na1	3a	0	0	0.137(22)	0.1(2)	3
O1	3a	0	0	0.408(7)	1	1
O2	3a	0	0	0.62(1)	1	1

Table S22 Atomic coordinates and isotropic thermal parameters (Biso, Å^2) of P3-$Na_{0.75}Mn_{0.68}Ni_{0.25}Zn_{0.07}O_2$ (Zn-P3) discharge to 3.8 V obtained from the Rietveld refinement shown in Figure S (c).

**Zn-P3 discharged 3.8 V**						
R_{wp}: 7.57%, R_{exp}: 6.41%, R_p: 5.55% Phase ratio: 57.63% Na ordered structure, 42.37% P3						
Lattice parameters Na ordered structure Space group *P121/m1* *a* = 4.955(2) Å, *b* = 5.733(2) Å, *c* = 5.957(3) Å, β = 106.39(2) °, V = 162.30(1) Å^3						
Atom	Wyckoff symbol	*x*/*a*	*y*/*b*	*z*/*c*	Occupancy	Biso/ Å^2
Mn1/Ni1/Zn1	3a	0	0	1/2	0.68/0.25/0/07	0.5
Mn2/Ni2/Zn2	3a	0.496(3)	1/4	0.493(2)	0.68/0.25/0/07	0.5
Na1	3a	0.365(7)	1/4	0.712(4)	0.51(4)	1
Na2	3a	0.197(7)	0	0.129(10)	0.22(2)	2
O1	3a	0.864(6)	1/4	0.676(6)	1	1
O2	3a	0.087(6)	1/4	0.327(6)	1	1
O3	3a	0.633(4)	0.015(4)	0.363(6)	1	1
Lattice parameters P3 Space group *R3m* *a* = 2.8584(9) Å, *c* = 17.032 (7) Å, V = 120.52 (9) Å^3						
Atom	Wyckoff symbol	*x*/*a*	*y*/*b*	*z*/*c*	Occupancy	Biso/ Å^2
Mn1/Ni1/Zn1	3a	0	0	0	0.68/0.25/0.07	0.5
Na1	3a	0	0	0.161(2)	0.54(4)	1
O1	3a	0	0	0.380(7)	1	1
O2	3a	0	0	0.623(7)	1	1

Table S23 Atomic coordinates and isotropic thermal parameters (Biso, Å^2) of P3-$Na_{0.75}Mn_{0.68}Ni_{0.25}Zn_{0.07}O_2$ (Zn-P3) discharge to 2.2 V obtained from the Rietveld refinement shown in Figure S (d).

**P3 $Na_{0.75}Mn_{0.68}Ni_{0.25}Zn_{0.07}O_2$ discharge 2.2 V**						
R_{wp}: 9.73%, R_p: 7.33%, R_{exp}: 7.30%						
Lattice parameters P3 Space group *R*3*m* *a* = 2.8944(14) Å, *c* = 16.771(17) Å, V = 121.68(17) Å^3						
Atom	Wyckoff symbol	*x*/*a*	*y*/*b*	*z*/*c*	Occupancy	Biso/ Å^2
Mn1/Ni1/Zn1	3a	0	0	0	0.68/0.25/0/07	0.42(9)
Na1	3a	0	0	0.171(2)	0.75(2)	7.1(6)
O1	3a	0	0	0.394(3)	1	1.5(2)
O2	3a	0	0	0.604(3)	1	1.5(2)

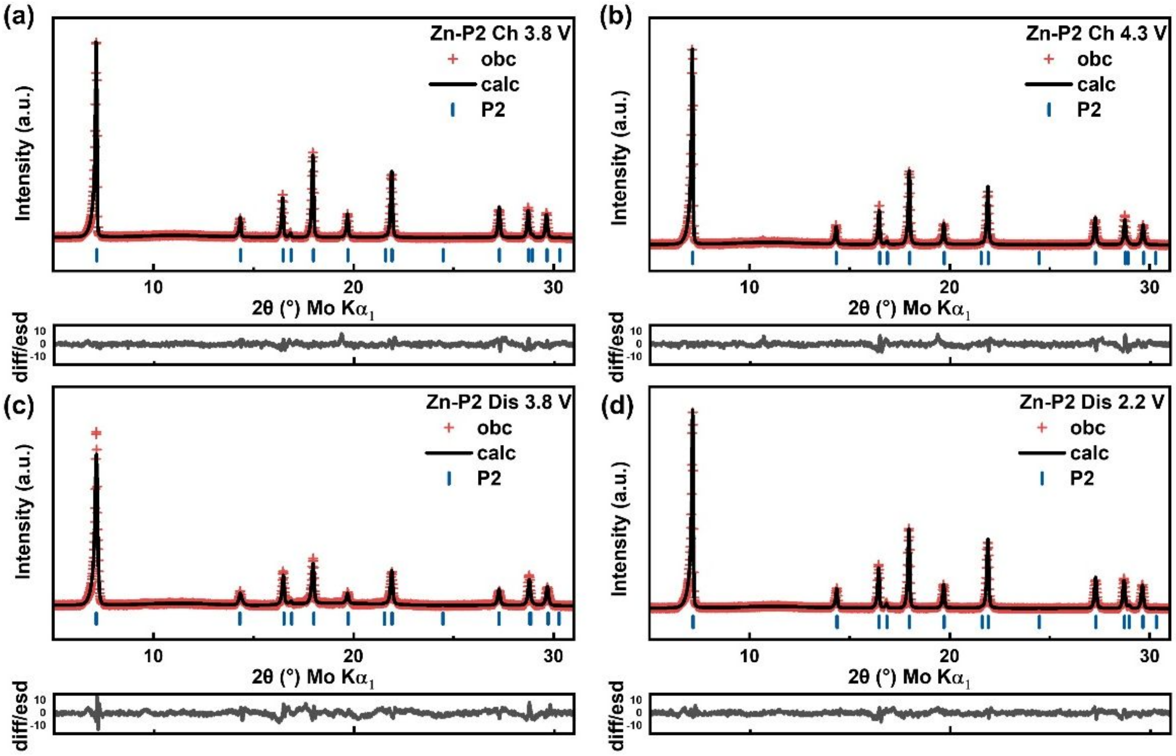

Figure S15 Laboratory X-ray Rietveld fits of Zn-P2 (a) charged to 3.8 V, (b) charged to 4.3 V, (c) discharged to 3.8 V after charge to 4.3 V and (d) discharge to 2.2 V after charge to 4.3 V. Observed data points are shown in red, with fitted profile in black.

Table S24 Atomic coordinates and isotropic thermal parameters (Biso, Å^2) of P2-$Na_{0.75}Mn_{0.68}Ni_{0.25}Zn_{0.07}O_2$ (Zn-P2) charge to 3.8 V obtained from the Rietveld refinement shown Figure S (a).

**P2 $Na_{0.75}Mn_{0.68}Ni_{0.25}Zn_{0.07}O_2$ charged 3.8V**						
R_{wp} : 8.02%, R_p : 5.69, R_{exp}: 6.40%<br>Lattice parameters P2 Space group *P*6$_3$/*mmc a* = 2.8567(2) Å, *c* = 11.3545(16) Å, V = 80.249 (16) Å^3						
Atom	Wyckoff symbol	*x*/*a*	*y*/*b*	*z*/*c*	Occupancy	Biso/ Å^2
Mn1/Ni1/Zn1	2*a*	0	0	0	0.68/0.25/0.07	0.72(3)
Na1	2*c*	0	0	1/4	0.10(1)	6(2)
Na2	2*b*	2/3	1/3	1/4	0.33(1)	6.0(6)
O1	4*f*	1/3	2/3	0.0871(4)	1	0.63(7)

Table S25 Atomic coordinates and isotropic thermal parameters (Biso, Å^2) of P2-$Na_{0.75}Mn_{0.68}Ni_{0.25}Zn_{0.07}O_2$ (Zn-P2) charge to 4.3 V obtained from the Rietveld refinement shown in Figure S (b).

**P2 $Na_{0.75}Mn_{0.68}Ni_{0.25}Zn_{0.07}O_2$ charged 4.3V**						
R_{wp}: 9.02%, R_{exp}: 6.59%, R_p: 6.55% Lattice parameters P2 Space group *P*6$_3$/*mmc* *a* = 2.8543(2) Å, *c* = 11.371(1) Å, V = 80.23 (1) Å^3						
Atom	Wyckoff symbol	*x*/*a*	*y*/*b*	*z*/*c*	Occupancy	Biso/ Å^2
Mn1/Ni1/Zn1	2*a*	0	0	0	0.68/0.25/0.07	0.76(3)
Na1	2*c*	0	0	1/4	0.09(8)	4
Na2	2*b*	2/3	1/3	1/4	0.224(6)	4
O1	4*f*	1/3	2/3	0.0847(5)	1	1.09(10)

Table S26 Atomic coordinates and isotropic thermal parameters (Biso, Å^2) of P2-$Na_{0.75}Mn_{0.68}Ni_{0.25}Zn_{0.07}O_2$ (Zn-P2) discharge to 3.8 V obtained from the Rietveld refinement shown in Figure S (c).

**P2 $Na_{0.75}Mn_{0.68}Ni_{0.25}Zn_{0.07}O_2$ discharge 3.8V**						
R_{wp}: 11.75%, R_{exp}: 6.36%, R_p: 8.78% Lattice parameters P2 Space group P6$_3$/mmc a = 2.8500(2) Å, c = 11.386(2) Å, V = 80.09 (2) Å^3						
Atom	Wyckoff symbol	*x*/*a*	*y*/*b*	*z*/*c*	Occupancy	Biso/ Å^2
Mn1/Ni1/Zn1	2*a*	0	0	0	0.68/0.25/0.07	0.5
Na1	2*c*	0	0	1/4	0.23	4
Na2	2*b*	2/3	1/3	1/4	0.3	8
O1	4*f*	1/3	2/3	0.0845(8)	1	0.8(2)

Table S270 Atomic coordinates and isotropic thermal parameters (Biso, Å^2) of P2-$Na_{0.75}Mn_{0.68}Ni_{0.25}Zn_{0.07}O_2$ (Zn-P2) discharge to 2.2 V obtained from the Rietveld refinement shown in Figure S (d).

**P2 $Na_{0.75}Mn_{0.68}Ni_{0.25}Zn_{0.07}O_2$ discharge 2.2V**						
R_{wp}: 8.13%, R_p: 5.70%, R_{exp}:5.31 %						
Lattice parameters P2 Space group *P*6$_3$/*mmc* *a* = 2.8951(4) Å, *c* = 11.157(3) Å, V = 80.99 (3) Å^3						
Atom	Wyckoff symbol	*x*/*a*	*y*/*b*	*z*/*c*	Occupancy	Biso/ Å^2
Mn1/Ni1/Zn1	2*a*	0	0	0	0.68/0.25/0.07	0.69(5)
Na1	2*c*	0	0	1/4	0.30(3)	7(1)
Na2	2*b*	2/3	1/3	1/4	0.49(3)	5.1(7)
O1	4*f*	1/3	2/3	0.0945(7)	1	0.9(1)

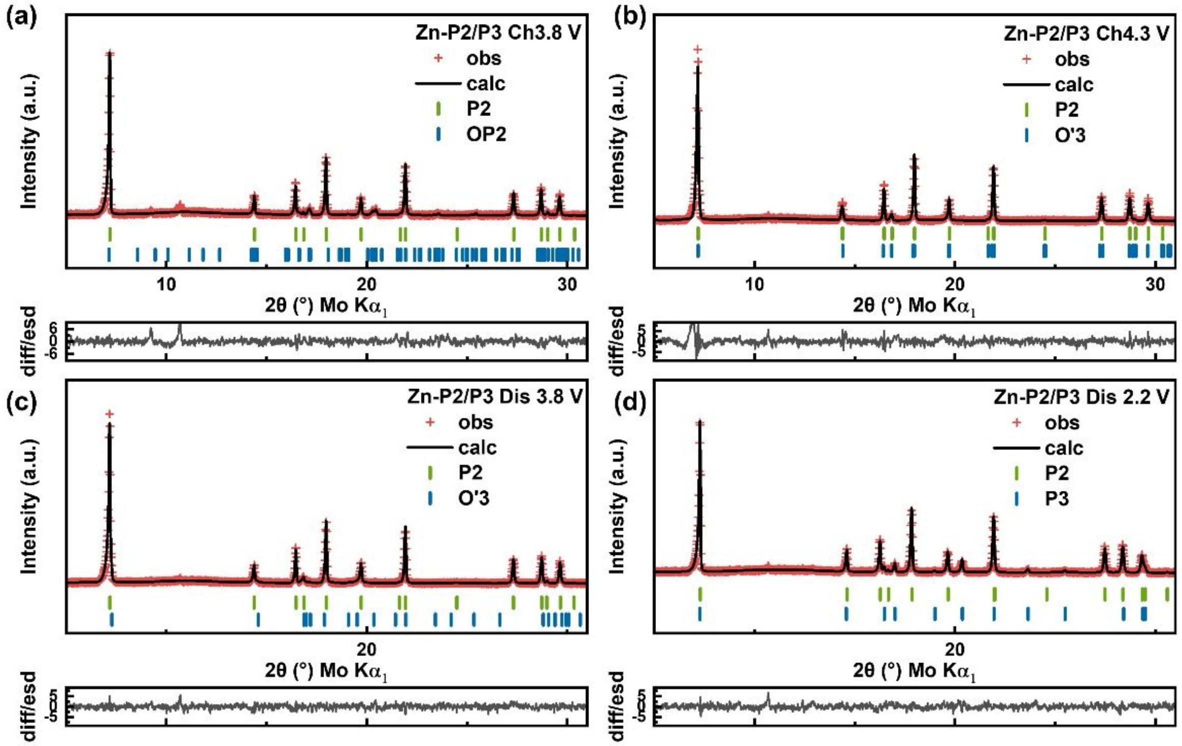

Figure S16 Laboratory X-ray Rietveld fits of Zn-P2/P3 (a) charged to 3.8 V, (b) charged to 4.3 V, (c) discharged to 3.8 V after charge to 4.3 V and (d) discharge to 2.2 V after charge to 4.3 V. Observed data points are shown in red, with fitted profile in black.

Table S28 Atomic coordinates and isotropic thermal parameters (Biso, Å^2) of P2/P3-$Na_{0.75}Mn_{0.68}Ni_{0.25}Zn_{0.07}O_2$ (P2/Zn-P3) charge to 3.8 V obtained from the Rietveld refinement shown in Figure S (a).

**P2/P3 $Na_{0.75}Mn_{0.68}Ni_{0.25}Zn_{0.07}O_2$ charge 3.8V**						
R_{wp}: 10.80%, R_p: 7.71%, R_{exp}: 9.31% Phase ratio: 86.08% P2, 13.92% OP2<br>Lattice parameters P2 Space group $P6_3/mmc$ a = 2.8606(2) Å, c = 11.324(2) Å, V = 80.25 (2) Å^3						
Atom	Wyckoff symbol	*x*/*a*	*y*/*b*	*z*/*c*	Occupancy	Biso/ Å^2
Mn1/Ni1/Zn1	2*a*	0	0	1/2	0.68/0.25/0.07	0.52(5)
Na1	2*c*	0	0	1/4	0.21 (3)	8(2)
Na2	2*b*	2/3	1/3	1/4	0.32(3)	6(1)
O1	4*f*	1/3	2/3	0.0890(7)	1	0.6(1)
Lattice parameters Na ordered structure Space group $P2_1/m$ a = 4.9565(1) Å, b= 5.725(1) Å, c = 5.930(2) Å, β = 106.68(2), V = 161.18(8) Å^3						

Table S29 Atomic coordinates and isotropic thermal parameters (Biso, Å^2) of P2/P3-$Na_{0.75}Mn_{0.68}Ni_{0.25}Zn_{0.07}O_2$ (P2/Zn-P3) charge to 4.3 V obtained from the Rietveld refinement shown in Figure S (b).

**P2/P3 $Na_{0.75}Mn_{0.68}Ni_{0.25}Zn_{0.07}O_2$ charge 4.3V**						
R_{wp}: 9.33%, R_{exp}: 9.59%, R_p: 6.91% Phase ratio: 86.97 % P2, 13.03% O'3<br>Lattice parameters P2 Space group $P6_3/mmc$ a = 2.8590(3) Å, c = 11.334(2) Å, V = 80.23 (2) Å^3						
Atom	Wyckoff symbol	*x*/*a*	*y*/*b*	*z*/*c*	Occupancy	Biso/ Å^2
Mn1/Ni1/Zn1	2*a*	0	0	1/2	0.68/0.25/0.07	0.66(4)
Na1	2*c*	0	0	1/4	0.14(2)	4(1)
Na2	2*b*	2/3	1/3	1/4	0.282(9)	2
O1	4*f*	1/3	2/3	0.0860(7)	1	0.36(10)
Lattice parameters O'3 Space group $C2/m$ a = 4.849(8) Å, b= 2.857 (3) Å, c = 5.91 (1) Å, β = 108.2(1), V = 77.7 (2) Å^3						
Atom	Wyckoff symbol	*x*/*a*	*y*/*b*	*z*/*c*	Occupancy	Biso/ Å^2
Mn1/Ni1/Zn1	3a	0	0	0	0.68/0.25/0.07	0.5
Na1	3a	0	0	1/2	0.10(7)	3
O1	3a	0.26(1)	0	0.142(7)	1	1

Table S30 Atomic coordinates and isotropic thermal parameters (Biso, Å^2) of P2/P3-$Na_{0.75}Mn_{0.68}Ni_{0.25}Zn_{0.07}O_2$ (P2/Zn-P3) discharge to 3.8 V obtained from the Rietveld refinement shown in Figure S (c).

**P2/P3 $Na_{0.75}Mn_{0.68}Ni_{0.25}Zn_{0.07}O_2$ discharge 3.8V**						
R_{wp}: 10.10%, R_p: 7.40%, R_{exp}: 9.31% Phase ratio: 85.93% P2, 14.07 % O'3						
Lattice parameters P2 Space group *P*6$_3$/*mmc* *a* = 2.8607(3) Å, *c* = 11.329(2) Å, V = 80.29 (2) Å^3						
Atom	Wyckoff symbol	*x*/*a*	*y*/*b*	*z*/*c*	Occupancy	Biso/ Å^2
Mn1/Ni1/Zn1	2*a*	0	0	1/2	0.68/0.25/0.07	0.6(1)
Na1	2*c*	0	0	1/4	0.10 (1)	4
Na2	2*b*	2/3	1/3	1/4	0.13(2)	4
O1	4*f*	1/3	2/3	0.0621(8)	1	1
Lattice parameters O'3 Space group *C2/m* *a* = 4.977(2) Å, *b*= 2.8618 (8) Å, *c* = 5.851 (2) Å, β = 107.67(3), V = 79.40 (5) Å^3						
Atom	Wyckoff symbol	*x*/*a*	*y*/*b*	*z*/*c*	Occupancy	Biso/ Å^2
Mn1/Ni1/Zn1	3a	0	0	0	0.68/0.25/0/07	0.2(2)
Na1	3a	0	0	1/2	0.38(4)	4
O1	3a	0.720(4)	0	0.237(4)	1	1

Table S31 Atomic coordinates and isotropic thermal parameters (Biso, Å^2) of P2/P3-$Na_{0.75}Mn_{0.68}Ni_{0.25}Zn_{0.07}O_2$ (P2/Zn-P3) discharge to 2.2 V obtained from the Rietveld refinement shown in Figure S (d).

**P2/P3 $Na_{0.75}Mn_{0.68}Ni_{0.25}Zn_{0.07}O_2$ discharge to 2.2V**						
R_{wp}: 9.77%, R_{exp}: 9.72%, R_p: 7.28% Phase ratio: 87.14% P2, 12.86% P3						
Lattice parameters P2 Space group $P6_3/mmc$ a = 2.8934(5) Å, c = 11.153(4) Å, V = 80.86 (4) Å^3						
Atom	Wyckoff symbol	*x/a*	*y/b*	*z/c*	Occupancy	Biso/ Å^2
Mn1/Ni1/Zn1	2*a*	0	0	1/2	0.68/0.25/0.07	0.60(5)
Na1	2*c*	0	0	1/4	0.26 (1)	6.1(5)
Na2	2*b*	2/3	1/3	1/4	0.51(2)	6.1(5)
O1	4*f*	1/3	2/3	0.0934(8)	1	0.7(1)
Lattice parameters P3 Space group $R3m$ a = 2.8923(6) Å, c = 16.7946 (73) Å, V = 121.67 (8) Å^3						
Atom	Wyckoff symbol	*x/a*	*y/b*	*z/c*	Occupancy	Biso/ Å^2
Mn1/Ni1/Zn1	3a	0	0	0	0.68/0.25/0/07	0.5
Na1	3a	0	0	0.174(2)	0.76(8)	1
O1	3a	0	0	0.390(7)	1	1
O2	3a	0	0	0.603(6)	1	1

Table S32 Summary of pair distribution function results for Zn-P2/P3 material.

		First neighbor in-plane (Å)		Second neighbor in-plane (Å)		Interlayer (Å)	
		M-O (d_1)	M-M(d_2)	M-O(d_3)	M-M(d_4)	M-M(d_5)	M-M(d_6)
Zn-P2/P3	Pristine	1.96	2.88	4.56	4.96	5.80	6.30
	Ch3.8V	1.92	2.84	4.54	4.92	5.78	6.32
	Ch4.3V	1.90	2.84	4.48	4.88	-	-
	Dis3.8V	1.90	2.86	4.50	4.92	5.72	6.30
	Dis2.2V	1.94	2.88	4.52	4.96	5.78	6.26

Reference

1 E. Flores, P. Novák and E. J. Berg, *Frontiers in Energy Research*, 2018, **6**, 1–16.
